# Proteolysis of HCF-1 by Ser/Thr glycosylation-incompetent *O*-GlcNAc transferase:UDP-GlcNAc complexes

**DOI:** 10.1101/gad.275925.115

**Published:** 2016-04-15

**Authors:** Vaibhav Kapuria, Ute F. Röhrig, Tanja Bhuiyan, Vladimir S. Borodkin, Daan M.F. van Aalten, Vincent Zoete, Winship Herr

**Affiliations:** 1 Center for Integrative Genomics, University of Lausanne, Lausanne 1015, Switzerland;; 2 Molecular Modeling Group, SIB Swiss Institute of Bioinformatics, Lausanne 1015, Switzerland;; 3 MRC Protein Phosphorylation and Ubiquitylation Unit, College of Life Sciences, University of Dundee, Dundee DD1 5EH, United Kingdom

**Keywords:** *O*-GlcNAcylation, post-translation modifications, proteolysis

## Abstract

In complex with the cosubstrate UDP-*N*-acetylglucosamine (UDP-GlcNAc), *O-*linked-GlcNAc transferase (OGT) catalyzes Ser/Thr *O*-GlcNAcylation of many cellular proteins and proteolysis of the transcriptional coregulator HCF-1. Such a dual glycosyltransferase–protease activity, which occurs in the same active site, is unprecedented and integrates both reversible and irreversible forms of protein post-translational modification within one enzyme. Although occurring within the same active site, we show here that glycosylation and proteolysis occur through separable mechanisms. OGT consists of tetratricopeptide repeat (TPR) and catalytic domains, which, together with UDP-GlcNAc, are required for both glycosylation and proteolysis. Nevertheless, a specific TPR domain contact with the HCF-1 substrate is critical for proteolysis but not Ser/Thr glycosylation. In contrast, key catalytic domain residues and even a UDP-GlcNAc oxygen important for Ser/Thr glycosylation are irrelevant for proteolysis. Thus, from a dual glycosyltransferase–protease, essentially single-activity enzymes can be engineered both in vitro and in vivo. Curiously, whereas OGT-mediated HCF-1 proteolysis is limited to vertebrate species, invertebrate OGTs can cleave human HCF-1. We present a model for the evolution of HCF-1 proteolysis by OGT.

## Abstract (precis)

In this study, Kapuria et al. investigate the dual glycosyltransferase–protease activity (which occurs in the same active site) of OGT. They show that glycosylation and proteolysis occur through separable mechanisms and present a model for the evolution of HCF-1 proteolysis by OGT.

Most eukaryotic proteins undergo post-translational modifications (PTMs), which diversify their function by regulating protein activity, localization, and turnover, thereby controlling diverse processes such as transcription, cell signaling, nutrient sensing, DNA repair, and apoptosis (Deribe et al. 2010; Hanover et al. 2012; Tessarz and Kouzarides 2014; Gambetta and Müller 2015). These PTMs can be both reversible (e.g., phosphorylation, ubiquitination, and glycosylation) and essentially irreversible (e.g., proteolytic cleavage). The finding (Capotosti et al. 2011) that the glycosyltransferase *O-*linked *N*-acetylglucosamine (*O*-GlcNAc) transferase (OGT) can both glycosylate cellular proteins and proteolytically cleave the transcriptional coregulator HCF-1 has presented an unforeseen intersection between reversible and irreversible protein modification.

*O-*GlcNAcylation involves the addition of GlcNAc to substrate Ser/Thr residues of numerous nuclear and cytoplasmic proteins (Hart et al. 2011). This reversible modification is achieved by a complex of OGT and the cosubstrate UDP-GlcNAc. As UDP-GlcNAc is produced by the nutrient-dependent hexosamine biosynthetic pathway (Hart et al. 2011), it has been suggested that *O*-GlcNAcylation by the OGT:UDP-GlcNAc complex serves as a nutrient sensor to regulate such processes as gene expression (see Dehennaut et al. 2014).

Human OGT consists of two distinct similarly sized domains: an N-terminal tetratricopeptide repeat (TPR) domain and a trilobal C-terminal catalytic (Cat) domain (Jinek et al. 2004; Lazarus et al. 2011). The TPR domain contains 13.5 TPRs, each representing a 34-amino-acid sequence that folds into two anti-parallel α helices; the 13.5 TPRs assemble to form a superhelical spiral fold (Jinek et al. 2004), a structure well suited to mediate protein–protein interactions (Kreppel and Hart 1999; Iyer and Hart 2003; Lazarus et al. 2013). The Cat domain contains N-terminal (N-Cat) and C-terminal (C-Cat) domains (separated by an intervening Int-D domain) with apparently different functions: The N-Cat domain contains residues implicated in catalysis, whereas the C-Cat domain provides binding sites for the sugar donor cosubstrate UDP-GlcNAc and sugar acceptor substrate peptide (Lazarus et al. 2011).

HCF-1 is an extensively *O*-GlcNAcylated transcriptional coregulator that, serving as an “adaptor” protein, links diverse DNA sequence-specific transcription factors with numerous different chromatin-modifying proteins, including OGT, and, as such, regulates mammalian cell proliferation and metabolism (Wysocka et al. 2003; Ruan et al. 2012; Zargar and Tyagi 2012). HCF-1 is produced as a large 2035-amino-acid precursor protein that undergoes OGT-mediated proteolysis at six centrally located, 26-amino-acid-long HCF-1 proteolytic (HCF-1_PRO_) repeats (Capotosti et al. 2011) to generate heterodimers of noncovalently associated N-terminal and C-terminal subunits (Kristie et al. 1995; Wilson et al. 1995; Park et al. 2012). OGT-mediated HCF-1 proteolysis is required for proper HCF-1 cell cycle regulation (Capotosti et al. 2011).

OGT-mediated HCF-1 proteolysis occurs N-terminal of a key glutamate residue at position 10 (E10) of the HCF-1_PRO_ repeat (in a C9–E10 sequence), creating a pyroglutamate “cap” on the C-terminal cleavage fragment (Lazarus et al. 2013). Cocrystallization of OGT and an HCF-1_PRO_ repeat revealed that the HCF-1_PRO_ repeat mimics the binding mode of a glycosylation-competent peptide by positioning the critical E10 residue at the same site as occupied by a serine glycosylation substrate (Lazarus et al. 2013). Indeed, an E-to-S substitution called E10S changes the HCF-1_PRO_ repeat from a proteolytic substrate into a glycosylation substrate, indicating a very close relationship between OGT-induced proteolysis and glycosylation (Lazarus et al. 2013).

Here, we investigated the mechanism of OGT-induced HCF-1 proteolysis by combining (1) computational modeling and simulation, (2) mutational approaches, and (3) chemical biology approaches. We show that, while at first appearing very similar, glycosylation and HCF-1 proteolysis are two distinguishable conserved activities of the OGT:UDP-GlcNAc complex.

## Results

### A flexible linker separates the cleavage and threonine-rich regions of the HCF-1_PRO_ repeat

**Figure 1.**
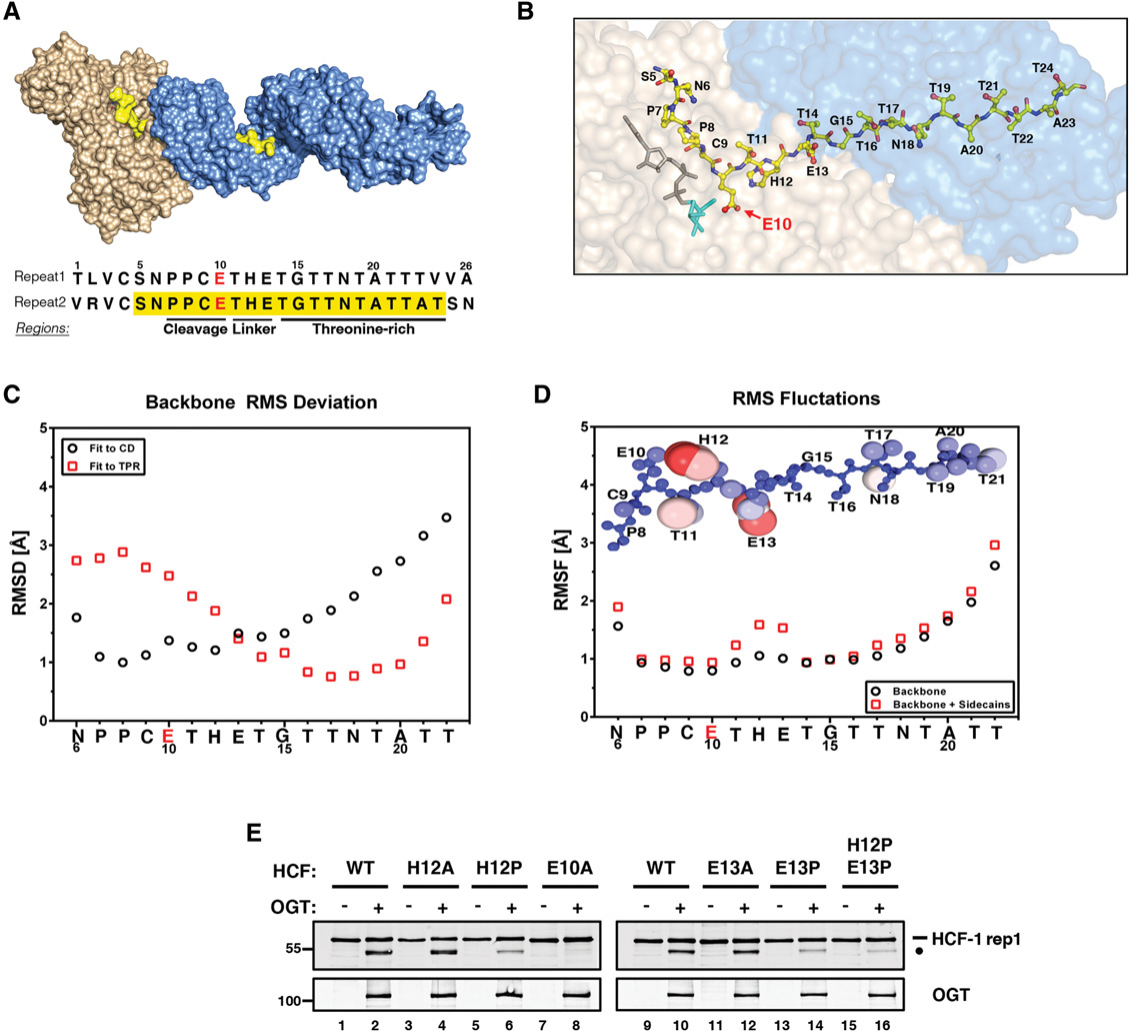
The HCF-1_PRO_ repeat contains a conserved flexible linker. (*A*) HCF-1_PRO_ repeat 2 (yellow) is shown bound to the TPR (blue) and Cat (tan) domains of full-length OGT. Sequences of highly conserved HCF-1_PRO_ repeats 1 and 2 are shown with the cleavage, linker, and threonine-rich regions indicated. Residues 5–24 of HCF-1_PRO_ repeat 2, observed in the crystal structure and used in the molecular dynamics (MD) simulations, are highlighted in yellow, while the critical cleavage site residue E10 is marked in red. (*B*) Depiction of HCF-1_PRO_ repeat 2 and OGT based on crystal structure 4N3B. The HCF-1_PRO_ repeat cleavage region (P7–E10) contacts the cosubstrate UDP-GlcNAc (shown in stick representation; [gray] UDP; [turquoise] GlcNAc) in the Cat domain (tan), whereas the threonine-rich region (T14–T24) contacts the OGT TPR domain (blue). We replaced the HCF-1_PRO_ repeat Q10 residue in the catalytically inactive crystal structure by E10 (red arrow) and replaced UDP-5S-GlcNAc by UDP-GlcNAc. (*C*) MD simulations identify correlations between HCF-1_PRO_ repeat 2 (residues 6–22) cleavage and threonine-rich region movements with UDP-GlcNAc-bound OGT Cat and TPR domains, respectively. The root mean square deviation (RMSD) of the HCF-1 backbone with respect to the Cat domain is depicted as black circles, while the RMSD with respect to the TPR domain is depicted as red squares. (*D*) Root mean square fluctuations (RMSFs) of the HCF-1_PRO_ repeat 2 (residues 6–22) bound to OGT. (Black circles) Backbone only; (red squares) backbone and side chains. (*Inset*) Structural representation of RMSFs illustrated by atom size and color (rigid is indicated by blue and small; flexible is indicated by red and large). (*E*) The HCF-1rep1 substrate (wild type [WT] or mutant) was assayed with or without OGT for in vitro HCF-1 cleavage activity. HCF-1rep1 cleavage was detected using anti-GST antibody, and OGT was detected using anti-T7 antibody. (Black bar) Uncleaved HCF-1rep1 substrate; (black circle) cleaved product.

The sequences of the six HCF-1_PRO_ repeats in human HCF-1 are highly similar (Wilson et al. 1993; Kristie et al. 1995). For historical and technical reasons and without any evident differences, studies of HCF-1_PRO_ repeat proteolysis by OGT have combined structural studies of OGT and HCF-1_PRO_ repeat 2 (Lazarus et al. 2013) and proteolysis studies of OGT and principally HCF-1_PRO_ repeat 1 (Capotosti et al. 2011; Lazarus et al. 2013). Figure 1A shows how these two HCF-1_PRO_ repeats are nearly identical in sequence as well as an illustration of the structure of HCF-1_PRO_ repeat 2 (yellow) bound to the OGT Cat (tan) and TPR (blue) domains prepared by combining two separate partial OGT crystal structures (Protein Data Bank 4N3B and 1W3B) (Jinek et al. 2004; Lazarus et al. 2011). As noted, the HCF-1_PRO_ repeat sequence has been subdivided into cleavage (defined here as P7–E10) and threonine-rich (T14–T24) regions (Capotosti et al. 2011). Figure 1B shows how the cleavage region, which spans the C9–E10 cleavage site, interacts with the Cat domain and the UDP-GlcNAc cosubstrate (gray and turquoise stick figures), whereas the threonine-rich region interacts with many residues in the TPR domain (Lazarus et al. 2013).

Based on the OGT:UDP-GlcNAc:HCF-1_PRO_ repeat 2 complex structure (Lazarus et al. 2013), we used molecular dynamics (MD) simulations to study the interactions between the HCF-1_PRO_ repeat cleavage and threonine-rich regions and the OGT Cat and TPR domains. In these simulations, we observed independent rigid body movements of the OGT Cat and TPR domains, as previously described (Lazarus et al. 2011). The movements of the cleavage region residues P7–E10 were strongly correlated with the OGT Cat domain (Fig. 1C, black circles), whereas the movements of the threonine-rich region residues T14–T22 were strongly correlated with the OGT TPR domain (Fig. 1C, red squares). HCF-1_PRO_ repeat residue E13, which lies between the cleavage and threonine-rich regions and at the interface of the Cat and TPR domains (see Fig. 1A,B), represents the crossover point between the observed Cat and TPR domain-associated movements.

MD simulation-derived thermal fluctuations of the backbone and side chains of OGT-bound HCF-1_PRO_ repeat 2 are displayed in Figure 1D. Whereas the HCF-1_PRO_ repeat 2 backbone generally showed small fluctuations (∼1.0 Å root mean square fluctuation [RMSF] for residues P7–E10 and T14–T19) (Fig. 1D, black circles), the side chains of residues T11, H12, and E13 showed significantly higher side chain fluctuations (∼1.5 Å RMSF) (Fig. 1D, red squares). These data are illustrated by the molecular representation in Figure 1D, where the atom size and color (small and blue represent rigid, and large and red represent flexible) reflect RMSF values. Together with the aforementioned backbone correlation data, these results suggest that a T11–H12–E13 “linker” sequence (see Fig. 1A) represents a flexible hinge between the cleavage and threonine-rich regions of the HCF-1_PRO_ repeat, allowing an optimal interaction with the positionally flexible Cat and TPR domains of OGT (see also Supplemental Fig. 1A).

To probe side by side the roles of side chain function and linker flexibility in HCF-1_PRO_ repeat cleavage experimentally, we investigated mutations of H12 and E13 in the previously described cleavage substrate HCF-1rep1 (Capotosti et al. 2011). Figure 1E shows the effects of mutations to alanine (H12A and E13A) to probe side chain function or to proline (individually H12P and E13P and in combination H12P/E13P) to restrict backbone movements and thus probe in part the role of flexibility. These mutations had no evident deleterious effect on HCF-1rep1 glycosylation (Supplemental Fig. 1B) or HCF-1_PRO_ repeat binding (Supplemental Fig. 1C). The alanine substitutions of H12 and E13 also had no evident effect on HCF-1_PRO_ repeat cleavage (Fig. 1E, cf. lanes 2, 4, 10, and 12), but the proline substitutions significantly inhibited cleavage (Fig. 1E, cf. lanes 2, 6, 10, 14, and 16). Although H12P and, to a lesser extent, E13P are predicted to have some structural impact on the OGT-bound HCF-1_PRO_ repeat (data not shown), these results are consistent with the “THE” linker sequence playing a flexibility as opposed to a side chain specificity role for proteolysis. These results support the hypothesis that the “THE” linker residues adapt the cleavage and threonine-rich regions of the HCF-1_PRO_ repeat to the motions of the OGT Cat and TPR domains.

### TPR motif requirements for OGT proteolysis of HCF-1

**Figure 2.**
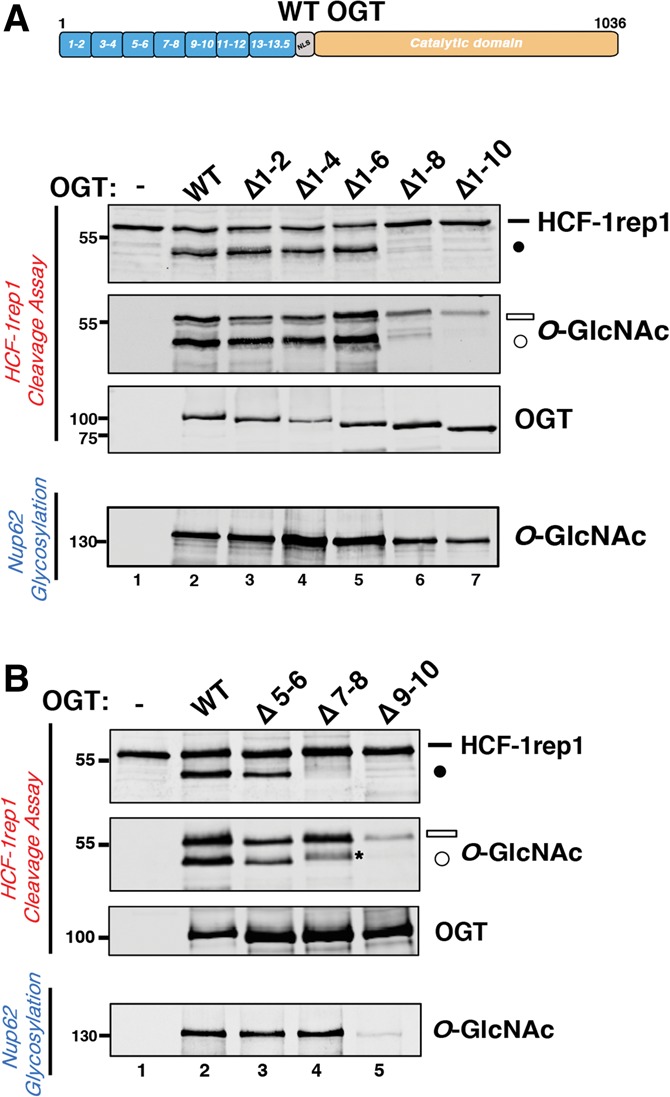
TPR domain requirements for HCF-1 proteolysis. (*A*) Mutant OGTs containing progressive N-terminal TPR deletions (schematic representation shown in Supplemental Fig. 2A) were incubated with HCF-1rep1 or Nup62 and assayed for in vitro HCF-1rep1 cleavage and glycosylation or Nup62 glycosylation as described in the Materials and Methods. HCF-1rep1 cleavage was detected with anti-GST antibody, whereas anti*-O-*GlcNAc RL2 was used to detect HCF-1rep1 and Nup62 glycosylation. Anti-T7 tag antibody was used to detect OGT. (*B*) Mutant OGTs containing internal deletions of two TPR motifs at a time (schematic representation shown in Supplemental Fig. 2B) were incubated with HCF-1rep1 or Nup62 and assayed for in vitro HCF-1rep1 cleavage and glycosylation or Nup62 glycosylation as described in *A*. The asterisk identifies a contaminating background band described in the Supplemental Material. (Black bar) Uncleaved HCF-1rep1 substrate; (black circle) cleaved product; (white bar) glycosylated uncleaved HCF-1rep1 substrate; (white circle) glycosylated cleaved product*.*

Next, we analyzed separately the function of the OGT TPR domain–HCF-1_PRO_ repeat threonine-rich region unit and the OGT Cat domain–HCF-1_PRO_ repeat cleavage region unit for glycosylation and HCF-1_PRO_ repeat cleavage by mutational analysis. With respect to the OGT TPR domain, the cocrystal structure of the HCF-1_PRO_ repeat with OGT illustrated for the first time in molecular detail how the TPR domain, in this case TPRs 10–13.5, can have a direct role in substrate binding and recognition (Lazarus et al. 2013). We therefore probed here, by deletion of TPR motif pairs, the overall role of the OGT TPR domain in proteolysis and glycosylation as shown in Figure 2. We first identified the minimal number of Cat domain-proximal OGT TPR motifs that is required for effective glycosylation and HCF-1_PRO_ repeat 1 cleavage by assaying the corresponding activities of a set of progressive N-terminal TPR motif pair deletion mutants. We used the HCF-1rep1 protein as a simultaneous glycosylation and cleavage substrate and used the well-described nuclear pore protein Nup62 substrate to assay glycosylation separately from cleavage (Lazarus et al. 2005, 2006), as shown in Figure 2A (see the deletion schematic in Supplemental Fig. 2A).

Deletion of up to six N-terminal TPR motifs in Δ1–2, Δ1–4, and Δ1–6 had no evident effect on either glycosylation or proteolysis of HCF-1rep1 (Fig. 2A, lanes 2–5); thus, the 7.5 Cat domain-proximal TPRs (TPRs 7–13.5) retain full glycosylation and HCF-1 proteolytic activities. In contrast, Δ1–8 and Δ1–10 exhibited a progressive loss of HCF-1rep1 and, to a lesser extent, Nup62 glycosylation activity (Fig. 2A, lanes 6,7), which was accompanied by a progressive increase in OGT autoglycosylation (see Supplemental Fig. 2A; Lubas and Hanover 2000; Clarke et al. 2008). In comparison, the Δ1–8 and Δ1–10 mutant OGT molecules were much less active for HCF-1rep1 proteolysis (Fig. 2A, lanes 6,7), suggesting a more significant role of OGT TPR motifs in HCF-1_PRO_ repeat cleavage than standard substrate glycosylation.

As the HCF-1_PRO_ repeat threonine-rich region makes multiple backbone and side chain contacts with Cat domain-proximal TPRs (Lazarus et al. 2013), we asked—by the creation of internal Δ5–6, Δ7–8, and Δ9–10 TPR deletion mutants—whether OGT TPR pairs play specific roles in HCF-1 proteolysis. As shown in Figure 2B, internal deletion of TPR pairs 5–6 and 7–8 had little effect on HCF-1rep1 or Nup62 glycosylation (lanes 2–4), whereas deletion of TPR pair 9–10 debilitated glycosylation of both substrates (lane 5) and activated OGT autoglycosylation (see Supplemental Fig. 2B, lane 5). (We note that the Δ9–10 deletion debilitated Nup62 glycosylation more than the Δ1–10 deletion [Fig. 2, cf. A, lane 7, and B, lane 5], suggesting that the remaining 1–8 TPRs in the Δ9–10 deletion have a dominant inhibitory effect in this mutant.) In comparison, the TPR pair deletion series had a more pronounced effect on HCF-1_PRO_ repeat proteolysis, as, albeit the Δ5–6 TPR deletion mutant was fully active, both the Δ7–8 and Δ9–10 TPR deletions displayed little if any HCF-1rep1 cleavage activity (Fig. 2B, lanes 3–5). The activity of the Δ5–6 TPR deletion mutant is consistent with the activity of the Δ1–6 TPR deletion. The inactivity of the Δ7–8 and Δ9–10 TPR deletion mutants suggests that the TPRs 7–10 play specific functional or overall structural roles for HCF-1rep1 cleavage. Consistent with this possibility, as illustrated in Supplemental Figure 2C, OGT TPR pairs differ considerably at multiple structurally exposed residues. Whichever the case, however, with both sets of TPR deletion mutants, HCF-1 proteolysis is more sensitive to OGT TPR deletions than is glycosylation of two different substrates.

### TPR mutations at the TPR–Cat domain interface disrupt HCF-1_PRO_ repeat cleavage without affecting Ser/Thr glycosyltransferase activity

**Figure 3.**
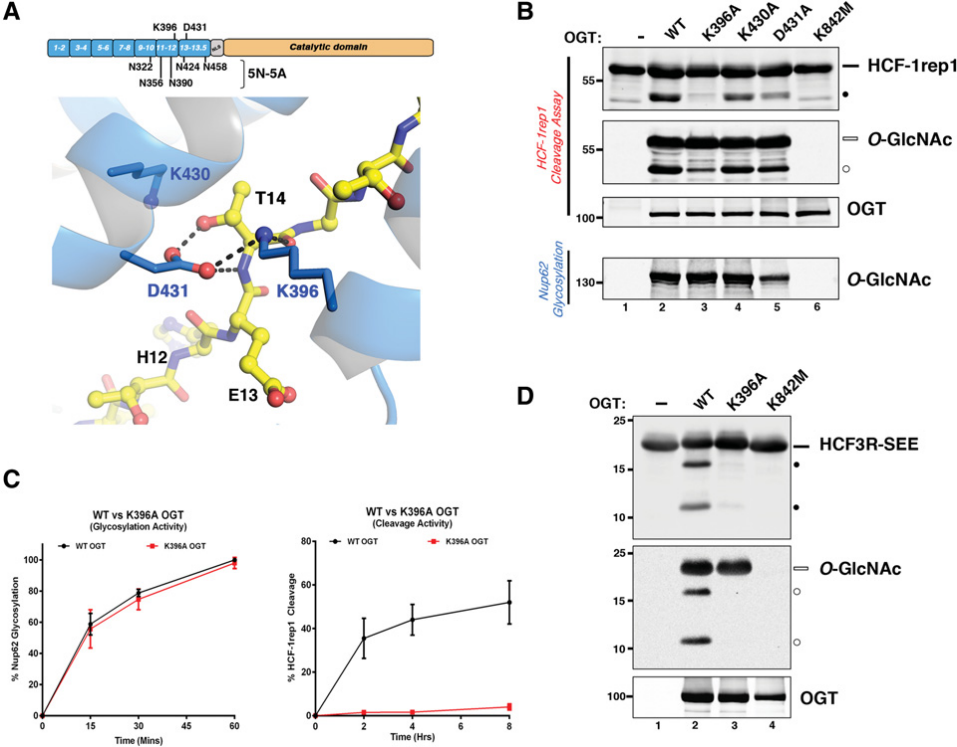
TPR domain point mutations at the TPR–Cat domain interface selectively impact HCF-1 proteolysis. (*A*, *top*) Domain representation of OGT: The positions of the five conserved asparagine residues replaced with alanines in the 5N-5A mutant (*below*) as well as residues K396 and D431 (*above*) are shown. Structural representation: HCF-1_PRO_ repeat 2 residue T14 interactions with K396 (TPR 12) and D431 (TPR 13) of the OGT TPRs. The OGT TPR domain and its residues are shown in blue, and the HCF-1_PRO_ repeat is shown as a yellow ball and stick representation. Interactions between T14 and OGT TPR residues are illustrated as black dashed lines. (*B*) HCF-1rep1 or Nup62 was incubated with wild-type or mutant OGTs and assayed for in vitro HCF-1 proteolysis and glycosylation or Nup62 glycosylation. HCF-1rep1 cleavage was detected with the anti-GST antibody, whereas HCF-1rep1 and Nup62 glycosylation was detected with the anti*-O-*GlcNAc RL2 antibody. Anti-OGT was used to detect OGT protein. (*C*) Graph depicting the Nup62 glycosylation (*left*) and HCF-1rep1 cleavage (*right*) activities of wild-type OGT and K396A OGT (Supplemental Fig. 3C,D). Glycosylation and cleavage efficiencies were calculated as described in the Materials and Methods. *n* = 3, ±SD. (*D*) HCF3R-SEE was incubated with wild-type, K396A, and K842M OGT to assay cleavage (*top* panel) and glycosylation (*middle* panel). Anti*-O-*GlcNAc RL2 antibody was used to detect HCF3R-SEE glycosylation, and anti-His and anti-OGT antibodies were used to detect total HCF3R-SEE and OGT protein, respectively. (Black bar) Uncleaved HCF-1rep1 substrate; (black circle) cleaved product; (white bar) glycosylated uncleaved HCF-1rep1 substrate; (white circle) glycosylated cleaved product*.*

To extend the analysis of the OGT TPR domain to those TPRs in the vicinity of the Cat domain, we took a point mutational approach. The HCF-1_PRO_ repeat threonine-rich region contacts five conserved asparagine residues of OGT TPRs 10–13.5, and simultaneous substitution of these asparagine residues for alanine (see Fig. 3A, labeled 5N-5A) disrupts HCF-1_PRO_ repeat–OGT interaction (Lazarus et al. 2013). The first threonine-rich region residue, T14 (see Fig. 1A), makes the first contacts with the OGT TPRs as the HCF-1_PRO_ repeat passes from the Cat domain to the TPR domain (Fig. 1B); there, it makes highly favorable peptide backbone contacts with the side chain of TPR 12 residue K396 and peptide backbone and side chain contacts with the side chain of TPR 13 residue D431 (Fig. 3A; Lazarus et al. 2013; Bhuiyan et al. 2015). Consistent with these contacts, T14 is important for HCF-1_PRO_ repeat cleavage (Wilson et al. 1995; Capotosti et al. 2011; Bhuiyan et al. 2015).

To probe further the importance of OGT TPR residues for both glycosylation and HCF-1rep1 cleavage, we mutated three OGT side chains in the vicinity of T14 (see Fig. 3A)—the previously mentioned K396 and D431 residues and K430, which does not contact the HCF-1_PRO_ repeat—and compared their activities with both wild-type OGT and the glycosylation-defective (Clarke et al. 2008) and proteolysis-defective (Lazarus et al. 2013) K842M OGT point mutant. All three OGT TPR point mutants retained HCF-1rep1 (Fig. 3B, HCF-1rep1 *O*-GlcNAc panel, lanes 2–5) and Nup62 (Fig. 3B bottom panel, lanes 2–5; see also Supplemental Fig. 3A) glycosyltransferase activity. (Note that the reduced Nup62 glycosylation activity of the D431A mutant observed in Figure 3B was exceptional to the assay shown and not reproducible.) In contrast, in the HCF-1rep1 cleavage assay, the three mutants were debilitated for cleavage activity, with the K396A mutant particularly so (Fig. 3B, top panel, lanes 3–5). The very weak K396A mutant cleavage activity was striking, and we therefore analyzed this mutant further.

First, we compared its relative HCF-1_PRO_ repeat cleavage and Nup62 glycosylation activity in time-course assays, as shown in Figure 3C (see Supplemental Fig. 3C,D for the primary data). These time courses revealed that the K396A OGT mutant possesses wild-type Nup62 glycosyltransferase activity (Fig. 3C left; see also casein kinase 2 [CK2] glycosylation in Supplemental Fig. 4A) but severely reduced (∼50-fold) HCF-1_PRO_ repeat cleavage activity (Fig. 3C, right). Thus, K396A OGT is an active glycosyltransferase with drastically reduced HCF-1_PRO_ repeat activity.

One reason for the absence of K396A OGT HCF-1 cleavage activity could be due to a loss of affinity for the HCF-1rep1 substrate, especially that the complementary HCF-1_PRO_ repeat T14A mutant substrate displays decreased OGT affinity (Bhuiyan et al. 2015). However, an in vitro binding assay showed that the K396A OGT mutant possesses good, albeit not wild-type, levels of HCF-1rep1-binding affinity—indeed, better affinity than the 5N-5A TPR mutant (Fig. 3A; Supplemental Fig. 3B, lanes 2–4); thus, the absence of K396A OGT proteolytic activity is not simply owing to a lack of affinity for the HCF-1_PRO_ repeat.

To compare directly the proteolysis-specific defect versus glycosylation activity of the K396A OGT mutant, we took advantage of the previously described single E10S HCF-1_PRO_ repeat amino acid exchange mutant by which (albeit artificially) the HCF-1_PRO_ repeat is converted from an OGT proteolysis substrate into a nearly identical Ser glycosyltransferase substrate (Lazarus et al. 2013).

To use this approach, we prepared a construct related to a previously described HCF3R construct, which contains HCF-1_PRO_ repeats 1–3 and thus is cleaved at three sites by OGT but serendipitously is not glycosylated by OGT (Lazarus et al. 2013). In our related construct called HCF3R-SEE, the HCF-1_PRO_ repeat 1 carries the E10S *O*-GlcNAc acceptor mutation. Using HCF3R-SEE, as shown in Figure 3D, the K396A OGT mutant again displays very weak proteolytic activity (top panel, cf. lanes 2–4) but, in the same reaction, robust E10S glycosylation activity, as evidenced by the effective *O*-GlcNAc labeling of the remaining uncleaved HCF3R-SEE precursor (middle panel, cf. lanes 2–4). Thus, the K396A substitution creates an OGT enzyme that is defective for proteolysis but not glycosylation at two sites that differ by only a single amino acid at the site of cleavage/glycosylation.

### Cat domain mutations can disrupt Ser/Thr glycosyltransferase activity without affecting HCF-1_PRO_ repeat cleavage

The above-mentioned studies illustrate how a bifunctional wild-type OGT capable of glycosylation and proteolysis can be converted (via mutation of the TPR domain) into a largely single-activity enzyme—in this case, readily active for Ser/Thr glycosylation but not HCF-1_PRO_ repeat proteolysis. By mutating the Cat domain, we show here how the opposite enzymatic preference can be achieved.

**Figure 4.**
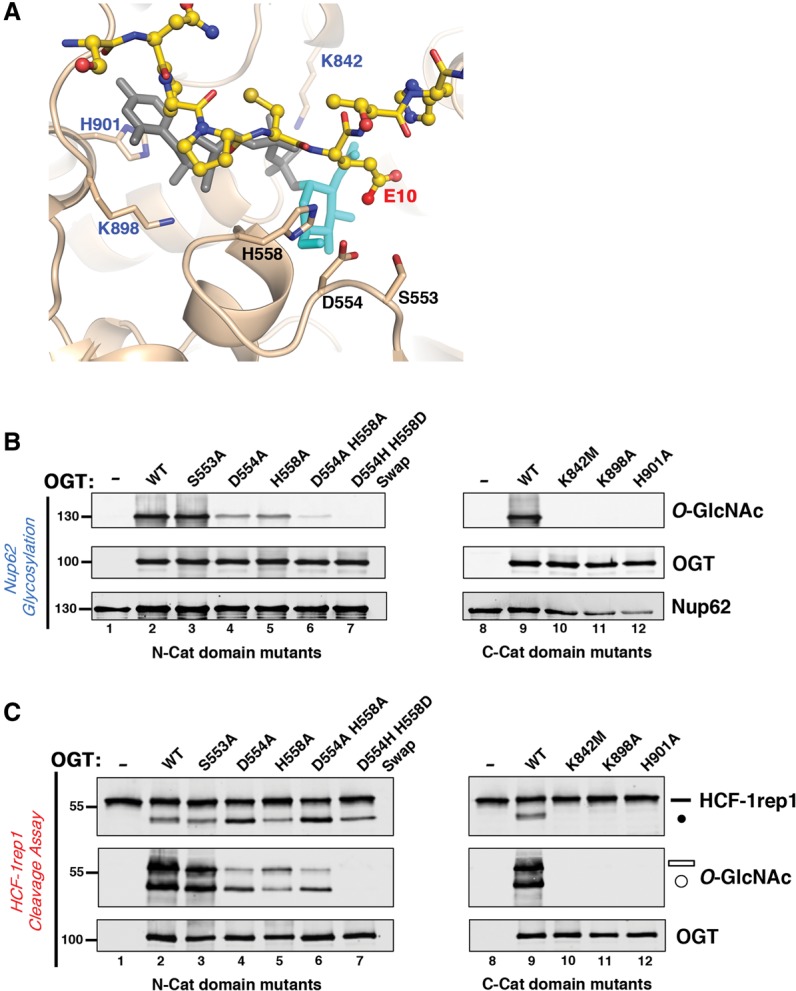
A glycosylation-defective OGT is effective for HCF-1 proteolysis. (*A*) Close-up view of the HCF-1_PRO_ repeat 2 cleavage region (yellow), with E10 (labeled in red) interacting with the UDP-GlcNAc-bound (gray–turquoise) OGT Cat domain (tan). N-Cat domain residues S553, D554, and H558 are labeled in black, whereas C-Cat domain residues K842, K898, and H901 that interact with the UDP-GlcNAc molecule are labeled in blue. (*B*) OGT N-Cat (lanes *3*–*7*) and C-Cat (lanes *10*–*12*) domain mutants were incubated with Nup62 to assay glycosylation activities. Anti*-O-*GlcNAc RL2 antibody was used to detect Nup62 glycosylation, whereas anti-OGT and anti-Nup62 antibodies were used to detect OGT and Nup62 protein levels, respectively. (*C*) HCF-1rep1 substrate was incubated with either wild-type or OGT N-Cat (lanes *3*–*7*) or C-Cat (lanes *10*–*12*) domain mutants for in vitro HCF-1rep1 cleavage and glycosylation assay. HCF-1rep1 cleavage and glycosylation were detected with anti-GST and anti*-O-*GlcNAc RL2 antibodies, respectively. Anti-OGT was used to detect OGT protein. (Black bar) Uncleaved HCF-1rep1 substrate; (black circle) cleaved product; (white bar) glycosylated uncleaved HCF-1rep1 substrate; (white circle) glycosylated cleaved product*.*

To date, manipulations of the UDP-GlcNAc cosubstrate and OGT C-Cat domain have shown parallel effects on glycosylation and HCF-1_PRO_ repeat cleavage. Thus, for both activities, UDP-GlcNAc is required (Capotosti et al. 2011; Lazarus et al. 2013), and the UDP-GlcNAc analog UDP-5S-GlcNAc (Gloster et al. 2011) is not active (Lazarus et al. 2013). Additionally, C-Cat domain mutations that block glycosylation also block HCF-1_PRO_ repeat cleavage (Capotosti et al. 2011; Lazarus et al. 2013). These findings suggest that OGT protein glycosylation and HCF-1_PRO_ repeat cleavage are closely related enzymatic activities. Consistent with these observations, as mentioned previously, the HCF-1 cleavage region mimics a glycosylation substrate when it binds to OGT (Lazarus et al. 2013). To probe the relationship between glycosylation and HCF-1_PRO_ repeat cleavage further, we assayed two series of Cat domain mutants (shown in Fig. 4A): one series involving residues K842 (Clarke et al. 2008; Martinez-Fleites et al. 2008), K898 (Clarke et al. 2008), and H901 (Lazarus et al. 2011) in the UDP-GlcNAc and substrate peptide-binding C-Cat domain (Lazarus et al. 2011) and a second series involving residues S553, D554 (Lazarus et al. 2005), and H558 (Martinez-Fleites et al. 2008; Lazarus et al. 2011) in the N-Cat domain critical for Ser/Thr substrate *O*-GlcNAcylation.

We first assayed the effect of these mutations on Nup62 substrate glycosylation. The three individual mutations within the C-Cat domain (K842M, K898A, and H901A) abrogated OGT Nup62 glycosylation activity (Fig. 4B, lanes 9–12). In the N-Cat domain, whereas the S553A OGT mutant had no apparent effect Fig. 4B, (lane 3), single alanine substitution of either of the neighboring D554 or H558 residues reduced Nup62 glycosylation activity (Fig. 4B, lanes 4,5), with the combined alanine substitutions (D554A_H558A) having a more severe effect (Fig. 4B, lane 6). Similar effects were observed for HCF-1rep1 glycosylation (Fig. 4C, middle panels).

The three glycosylation-defective C-Cat domain mutations were also defective for HCF-1_PRO_ repeat cleavage (Fig. 4C, top panel, lanes 9–12). In contrast, the N-Cat domain D554, H558, and D554A_H558A glycosylation-debilitated mutants retained wild-type HCF-1_PRO_ repeat cleavage activity (Fig. 4C, lanes 4–6); indeed, the D554A mutation in isolation or in combination with the H558A mutation enhanced HCF-1_PRO_ repeat cleavage. These results indicate that these latter residues are more important for Ser/Thr glycosylation than proteolysis.

This observation is strikingly reinforced by the activity of a mutation in which the D554 and H558 side chains have been exchanged in a so-called D554H_H558D “swap” mutant. Here, the D554H_H558D swap mutant did not display either Nup62 (Fig. 4B, lane 7; see also Supplemental Fig. 4A for CK2 glycosylation) or HCF-1rep1 glycosylation activity but did display full HCF-1_PRO_ repeat cleavage activity (Fig. 4C, lane 7). Consistent with the wild-type level of proteolytic activity, MD simulations failed to predict any effect of the D554H_H558D swap mutant on the global structure of OGT or on the binding of the HCF-1_PRO_ repeat (Supplemental Fig. 4B). These results show that, in contrast to the TPR domain mutations, OGT N-Cat domain mutations can strongly reduce Ser/Thr glycosyltransferase activity (see also Bhuiyan et al. 2015) without evidently affecting HCF-1_PRO_ repeat cleavage activity. Additional OGT Cat domain mutants, which showed either no inhibition of HCF-1rep1 proteolytic and glycosylation activity or a concomitant decrease of both activities, are described in Supplemental Figure 4C.

### HCF-1 proteolysis is independent of the UDP-GlcNAc α-phosphate oxygen-based Ser/Thr glycosylation mechanism

Schimpl et al. (2012) proposed that the α-phosphate of the UDP-GlcNAc plays a direct role in OGT-mediated substrate *O*-GlcNAcylation by activating the acceptor site Ser/Thr side chain. Using diastereomers of αS-UDP-GlcNAc in which either one or the other nonbonded α-phosphate oxygen atoms (pro-S [S_p_] and pro-R [R_p_]) is uniquely replaced with a sulfur atom, they showed that only the S_p_-αS-UDP-GlcNAc diastereomer, where the oxygen faces the acceptor Ser/Thr residue, is functional for glycosylation. Here, we asked whether the same αS-UDP-GlcNAc diastereomer selectivity would be observed in HCF-1_PRO_ repeat cleavage.

**Figure 5.**
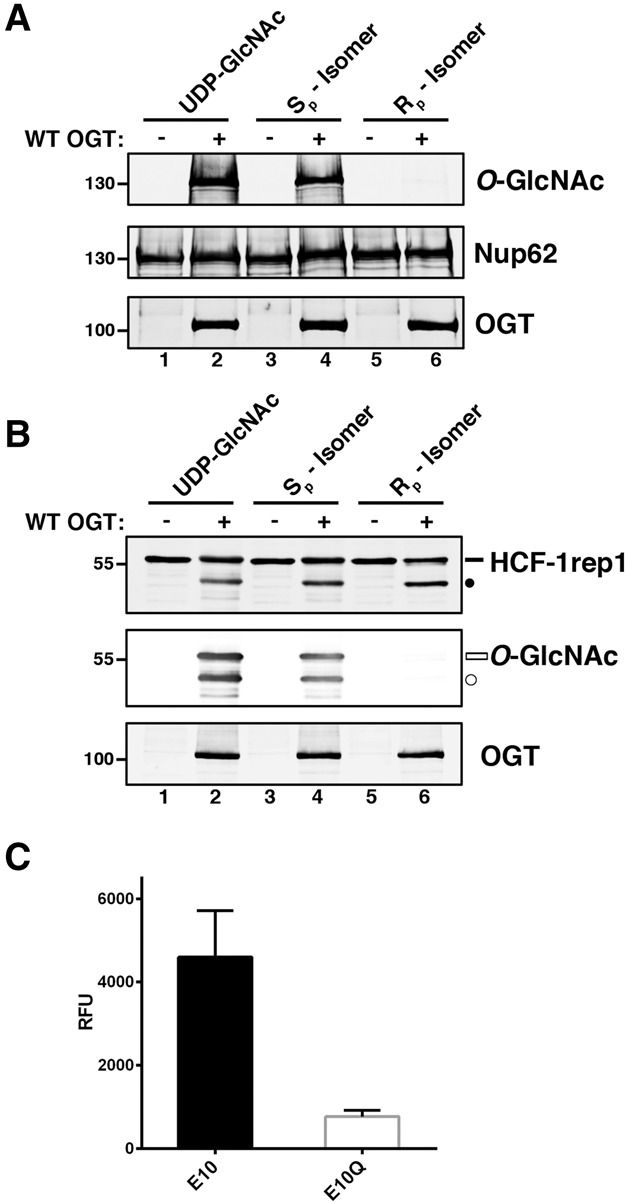
HCF-1 cleavage occurs independently of an α-phosphate-based mechanism. (*A*) Nup62 glycosylation with (lanes *1*,*3*,*5*) or without (lanes *2*,*4*,*6*) wild-type OGT and either UDP-GlcNAc (lanes *1*,*2*), S_p_-αS-UDP-GlcNAc (lanes *3*,*4*), or R_p_-αS-UDP-GlcNAc (lanes *5*,*6*). Nup62 glycosylation was detected using anti*-O-*GlcNAc RL2 antibody. Anti-Nup62 and anti-OGT antibodies were used to detect Nup62 and OGT proteins, respectively. (*B*) HCF-1rep1 cleavage assay with (lanes *1*,*3*,*5*) or without (lanes *2*,*4*,*6*) wild-type OGT and either UDP-GlcNAc (lanes *1*,*2*), S_p_-αS-UDP-GlcNAc (lanes *3*,*4*), or R_p_-αS-UDP-GlcNAc (lanes *5*,*6*). HCF-1rep1 cleavage and glycosylation were detected using anti*-*GST and anti*-O-*GlcNAc RL2 antibodies, respectively. Anti-OGT antibody was used to detect OGT protein levels. (*C*) UDP released as a by-product of HCF-1rep1 cleavage assays with R_p_-αS-UDP-GlcNAc with wild-type or uncleavable E10Q HCF-1rep1 substrates was detected fluorimetrically, as described in the Materials and Methods. *n* = 3, ±SD. (Black bar) Uncleaved HCF-1rep1 substrate; (black circle) cleaved product; (white bar) glycosylated uncleaved HCF-1rep1 substrate; (white circle) glycosylated cleaved product.

Consistent with prior experiments, which used Tab1 as substrate (Schimpl et al. 2012), the S_p_-αS-UDP-GlcNAc diastereomer but not the R_p_-αS-UDP-GlcNAc diastereomer was functional for both Nup62 (Fig. 5A, cf. lanes 4 and 6) and HCF-1rep1 (Fig. 5B, middle panel, lanes 4,6) glycosylation by OGT. In stark contrast, both αS-UDP-GlcNAc-diastereomers were functional for HCF-1_PRO_ repeat cleavage (Fig. 5B, top panel, lanes 4,6). This differential requirement for the UDP-GlcNAc α-phosphate R_p_ oxygen for Ser/Thr glycosylation and HCF-1_PRO_ repeat cleavage shows that UDP-GlcNAc is used differently for Ser/Thr glycosylation and proteolysis. Together with the selective proteolytic activity of the D554H_H558D swap mutant described above, these results show that both components of the OGT:UDP-GlcNAc complex are differentially involved in Ser/Thr glycosylation and HCF-1_PRO_ repeat cleavage.

The above-mentioned results indicate that the requirements for the OGT Cat domain and UDP-GlcNAc in HCF-1_PRO_ repeat cleavage are less stringent than for Ser/Thr glycosylation. The unexpected activity of the OGT:R_p_-αS-UDP-GlcNAc complex for HCF-1_PRO_ repeat cleavage led us to ask whether UDP-GlcNAc is used during the HCF-1_PRO_ repeat cleavage reaction when the R_p_-αS-UDP-GlcNAc diastereomer is used by assaying UDP release using a recently developed UDP sensor compound (see the Materials and Methods; Borodkin et al. 2014). Figure 5C shows that the wild-type HCF-1rep1 cleavage substrate (E10) generated UDP in the presence of the R_p_-αS-UDP-GlcNAc diastereomer considerably more effectively than the noncleavable HCF-1rep1 E10Q point mutant. Thus, the R_p_ oxygen is not required for HCF-1_PRO_ repeat cleavage, but UDP-GlcNAc is still consumed during the cleavage reaction.

### Altered Ser/Thr glycosylation and HCF-1_PRO_ repeat cleavage activities are reproduced in an in vivo assay

**Figure 6.**
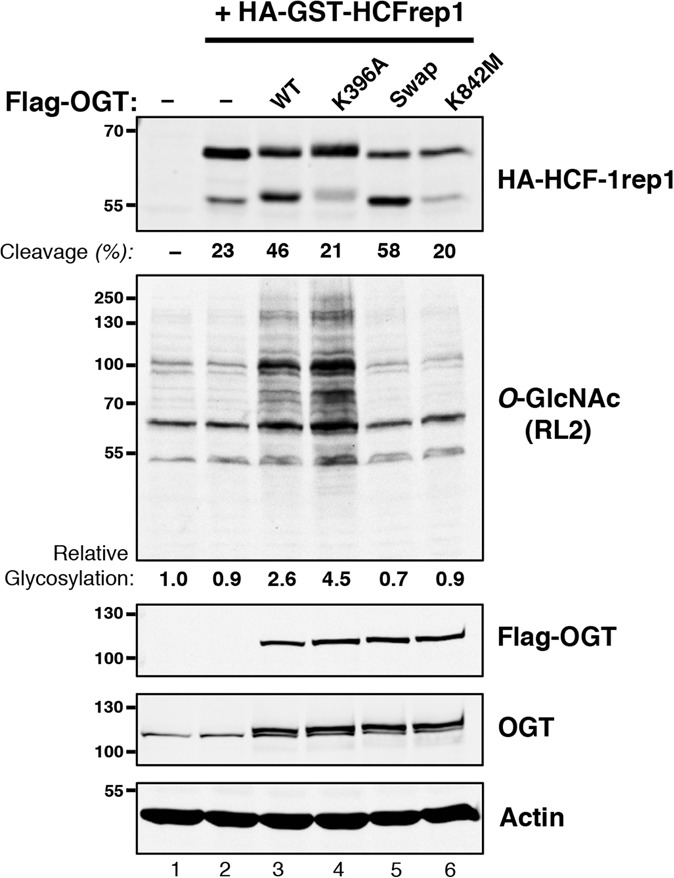
In vivo glycosylation and HCF-1rep1 cleavage properties of activity-selective OGT mutants. HEK293 cells were untransfected (lane *1*), transfected with HA-GST–HCF-1rep1 vector alone (lane *2*), cotransfected with wild-type (lane *3*) or mutant (lanes *4*–*6*) Flag-OGT expression vectors as described in the Materials and Methods. (*Top* panel) HA-GST–HCF-1rep1 cleavage was detected using anti-HA antibody and quantified as described in the Materials and Methods. Endogenous HEK293 protein *O*-GlcNAcylation was visualized with anti-*O-*GlcNAc RL2 antibody. Levels of ectopic recombinant Flag-OGT were detected alone or in combination with endogenous OGT with anti-Flag or anti-OGT antibody, respectively. (*Bottom* panel) An anti-actin blot is shown as a loading control. To quantitate *O*-GlcNAcylation of endogenous proteins, the total combined intensity of *O*-GlcNAcylated proteins in the untransfected HEK29 cells was assigned an arbitrary value of 1. The relative intensities of *O*-GlcNAcylation in each transfected sample were then calculated as a ratio between its combined protein *O-*GlcNAcylation intensity over that of the untransfected lysate. The quantitation indicated is for the experiment shown. Similar results were obtained in three additional separate experiments.

The above-mentioned studies have revealed that the in vitro Ser/Thr glycosyltransferase and HCF-1_PRO_ repeat cleavage activities of OGT can be effectively separated by point mutation. We asked here whether these selective effects are maintained in an in vivo environment, as we imagined, for example, that cellular chaperones or other OGT-associated proteins might maintain lost in vitro activities and/or modify altered activities of selectively active mutant OGT molecules. For this purpose, we developed an in vivo assay by which the activity of ectopically synthesized wild-type and mutant OGT molecules can be assayed for both Ser/Thr glycosylation of endogenous proteins and cleavage of a cosynthesized HCF-1rep1 substrate as shown in Figure 6.

Ectopic synthesis of HCF-1rep1 molecules in HEK293 cells results in partial cleavage owing to the activity of endogenous OGT (Bhuiyan et al. 2015). To establish our assay, we showed that coectopic synthesis of wild-type OGT (Fig. 6, Flag-OGT and OGT panels, lane 3) results in an enhanced cleavage of a cosynthesized HCF-1rep1 substrate (Fig. 6, top panel, cf. lanes 2 and 3) and an enhanced glycosylation of endogenous proteins (Fig. 6, *O*-GlcNAc panel, cf. lanes 2 and 3). We note that with enhanced HCF-1rep1 cleavage, there is also a shift in the mobility of the uncleaved and cleaved molecules; we suggest that this mobility shift is due to increased glycosylation of HCF-1rep1 molecules by the elevated levels of wild-type OGT. Consistent with such an interpretation, overexpression of the inactive K842M OGT mutant did not affect either HCF-1rep1 proteolysis or endogenous protein glycosylation and did not induce an HCF-1rep1 fragment mobility shift (Fig. 6, lane 6).

Reflective of their in vitro activities, in vivo, the Ser/Thr glycosylation-competent K396A OGT mutant failed to enhance HCF-1rep1 cleavage but did enhance glycosylation of endogenous proteins and induce an HCF-1rep1 fragment mobility shift (Fig. 6, lane 4), and the proteolysis-competent swap OGT mutant enhanced HCF-1rep1 cleavage but not glycosylation of endogenous proteins and did not induce an HCF-1rep1-fragment mobility shift. Thus, the selective Ser/Thr glycosylation and proteolytic activities of the K396A and swap OGT mutants observed in vitro are retained in an in vivo environment.

We note with interest that, in the assay of the swap OGT mutant, the glycosylation status of none of the endogenous proteins was evidently enhanced using two different *O*-GlcNAc antibodies (see Supplemental Fig. 5). This result suggests that the Ser/Thr glycosylation defect of the swap OGT mutant is not substrate-specific.

### The ability to cleave the HCF-1_PRO_ repeat is conserved in invertebrate OGT molecules

**Figure 7.**
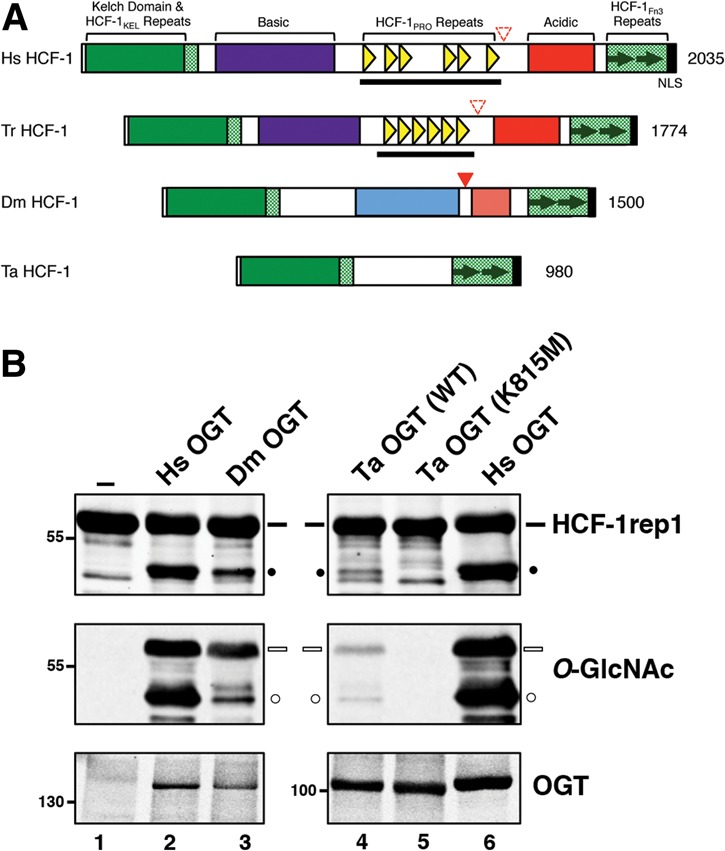
Invertebrate OGTs are active for HCF-1_PRO_ repeat cleavage. (*A*) Human (*Homo sapiens* [Hs]) HCF-1 and its homologs found in other vertebrate (fish *Takifugu rubripres* [Tr]) and invertebrate (*Drosophila melanogaster* [Dm] and *Trichoplax adhaerens* [Ta]) species are shown. The two vertebrate HCF-1 proteins contain HCF-1_PRO_ repeats (yellow triangles), which are coded by single large exons (black bar). (Red filled triangle) Taspase 1 cleavage site; (red outlined triangle) degenerate Taspase 1 cleavage site; (Fn3) fibronectin type 3; (NLS) nuclear localization signal. (*B*) HCF-1rep1 cleavage assay was performed using human (lanes *2*,*6*), wild-type and K815M mutant *Trichoplax* (lanes *3*,*4*, respectively), and *Drosophila* (lane *7*) OGTs as described in the Materials and Methods. HCF-1rep1 cleavage and glycosylation were detected using anti*-*GST and anti*-O-*GlcNAc RL2 antibodies, respectively. Anti-his tag antibody was used to detect OGT protein levels. (Black bar) Uncleaved HCF-1rep1 substrate; (black circle) cleaved product; (white bar) glycosylated uncleaved HCF-1rep1 substrate; (white circle) glycosylated cleaved product.

OGT glycosylation activity is conserved among both vertebrates and invertebrates (see OGT sequence comparison in Supplemental Fig. 6; Hanover et al. 2005; Gambetta et al. 2009; Sinclair et al. 2009; Selvan et al. 2015). HCF-1 is also conserved, but, as shown for humans and fish in Figure 7A, the HCF-1_PRO_ repeat is present in HCF-1 only among vertebrate species. In contrast, in some invertebrate species (e.g., *Drosophila*), the HCF-1 homolog is still cleaved but by a different protease called Taspase 1 (Capotosti et al. 2007). Thus, HCF-1 proteolysis occurs in both vertebrates and invertebrates, but only in vertebrates is the unusual ability of OGT to cleave the HCF-1_PRO_ repeat used for HCF-1 cleavage. This observation raises the question: Did vertebrate OGTs evolve to cleave the HCF-1_PRO_ repeat, or, instead, did the HCF-1_PRO_ repeat evolve to be cleaved generally by OGTs?

To address this question, we tested the ability of two invertebrate OGT proteins—that of *Drosophila melanogaster*, where HCF-1 is cleaved by Taspase 1 (Capotosti et al. 2007), and that of *Trichoplax adhaerens* (Selvan et al. 2015), where HCF-1 lacks an evident proteolytic cleavage site (see Fig. 7A)—to cleave the HCF-1_PRO_ repeat. Indeed, as shown in Figure 7B, both *D. melanogaster* OGT (lane 3) and *T. adhaerans* OGT (lane 4) were active for HCF-1rep1 glycosylation and cleavage (see the black dots), with *D. melanogaster* OGT being more active than *T. adhaerans* OGT. Although less active, the veracity of *T. adhaerans* OGT cleavage of the HCF-1_PRO_ repeat was supported by the finding that a *T. adhaerans* OGT K815M mutation corresponding to the Ser/Thr glycosylation and HCF-1_PRO_ repeat cleavage-defective human OGT K842M mutation (see Fig. 3) was also defective for HCF-1_PRO_ repeat cleavage (Fig. 3, cf. lanes 4 and 5). Consistent with conserved invertebrate HCF-1 cleavage activity, residues that are involved in HCF-1 proteolysis are conserved in invertebrate OGT homologs (Supplemental Fig. 6). Of particular interest here is the residue K396 because we show that, while important for proteolysis, it is not necessary for Ser/Thr glycosylation and yet is conserved. Nevertheless, apparently, OGT did not evolve to cleave the HCF-1_PRO_ repeat, but rather the HCF-1_PRO_ repeat evolved to be cleaved by OGT.

## Discussion

In this study, we separated the requirements for *O*-linked Ser/Thr glycosylation and site-specific HCF-1_PRO_ repeat cleavage by the glycosyltransferase–protease OGT, thus engineering, from a bifunctional enzyme, monofunctional OGT:UDP-GlcNAc complexes fully active for only *O*-linked Ser/Thr glycosylation or HCF-1_PRO_ repeat proteolysis in vitro or in vivo. The domains responsible for supporting monofunctionality differed: Mutations in the OGT TPR domain could affect HCF-1_PRO_ repeat cleavage without affecting Ser/Thr glycosylation (see Figs. 2, 3), and either (1) mutations in the OGT Cat domain or (2) chemical alterations of UDP-GlcNAc could affect Ser/Thr glycosylation without affecting HCF-1_PRO_ repeat cleavage (see Figs. 4, 5). As discussed below, we suggest (1) that the OGT TPR domain is more critical for positioning the HCF-1_PRO_ repeat for cleavage than it is for positioning many Ser/Thr glycosylation substrates and (2) that the OGT Cat domain and UDP-GlcNAc are more critical for Ser/Thr glycosylation than for HCF-1_PRO_ repeat cleavage.

The HCF-1_PRO_ repeat threonine-rich region makes numerous intimate contacts within the OGT TPR domain, whereas the HCF-1_PRO_ repeat cleavage region interacts with the OGT Cat domain and UDP-GlcNAc cosubstrate (Lazarus et al. 2013). OGT MD simulations have previously indicated large movements of the TPR and Cat domains around a TPR 12–13 hinge (Lazarus et al. 2011). Here, intimate HCF-1_PRO_ repeat and OGT interactions are highlighted by the tightly coupled movements of the HCF-1_PRO_ repeat threonine-rich region with the OGT TPR domain and the HCF-1_PRO_ repeat cleavage region with the OGT Cat domain. As revealed by MD simulations and mutational analyses (see Fig. 1), these intimate HCF-1_PRO_ repeat–OGT interactions are aided by a novel “flexibility” role of the small three-amino-acid “THE” linker region, which adapts the HCF-1_PRO_ repeat to the bipartite OGT movements, promoting HCF-1 peptide backbone flexibility that is important for its efficient cleavage.

Previous studies have shown that mutant OGTs containing truncations of its N-terminal TPR domain are still active for peptide glycosylation (Iyer and Hart 2003). The TPR domain deletion analysis presented here suggests that OGT's Ser/Thr glycosylation activity is less sensitive to progressive N-terminal deletion than its HCF-1_PRO_ repeat cleavage activity (see Fig. 2). Indeed, fully 6.5–7.5 Cat domain-proximal TPR motifs are critical for efficient HCF-1_PRO_ repeat cleavage but not substrate glycosylation. Interestingly, the HCF-1_PRO_ repeat is the only OGT substrate thus far crystallized that shows intimate binding with the extended OGT TPR domain (Lazarus et al. 2013); other OGT glycosylation substrates only extend until the TPR–Cat domain interface (Lazarus et al. 2011; Pathak et al. 2015). Within the Cat-proximal 7.5 TPR motifs, there are stringent TPR motif sequence requirements for HCF-1_PRO_ repeat cleavage, as, for example, HCF-1_PRO_ repeat cleavage but not Ser/Thr glycosylation is sensitive to internal deletion of TPRs 7 and 8 in this region. Consistent with these findings is the special nature of the K396A mutation in TPR 12, which generates a Ser/Thr glycosyltransferase-competent but proteolysis-defective enzyme. The ability of the K396A OGT mutant to bind but not cleave the HCF-1_PRO_ repeat substrate suggests that, in addition to binding the substrate, the TPR domain plays a specific role in HCF-1_PRO_ repeat cleavage; we suggest that this role is in correctly “positioning” the HCF-1_PRO_ repeat cleavage region in the Cat domain for cleavage. This function could be critical, as the key E10 glutamate for proteolysis specifically inhibits HCF-1_PRO_ repeat binding to the Cat domain (Bhuiyan et al. 2015).

Within the OGT C-Cat domain responsible for UDP-GlcNAc binding, deleterious mutations affect both Ser/Thr glycosylation and HCF-1_PRO_ repeat cleavage (see Fig. 4; Supplemental Fig. 4; Capotosti et al. 2011; Lazarus et al. 2013; this study), suggesting that the binding and positioning of UDP-GlcNAc in the Cat domain are critical for both activities. In contrast, N-Cat domain mutations can reduce Ser/Thr glycosylation activity (Lazarus et al. 2013) without, as shown here, affecting proteolysis. Here, we further identified the so-called N-Cat domain D554H_H558D swap mutant, which dramatically impairs Ser/Thr glycosylation without having any evident adverse effect on proteolysis. This distinction between glycosylation and proteolysis is now extended by the activities of the R_p_-αS-UDP-GlcNAc-bound OGT, which likewise, while failing to glycosylate Nup62 or other proteins, is still fully active for HCF-1_PRO_ repeat cleavage.

Thus, with respect to both components of the OGT:UDP-GlcNAc complex, we observed effective HCF-1_PRO_ repeat cleavage by glycosylation-incompetent enzyme:cosubstrate complexes. A model has suggested that HCF-1 proteolysis is initiated through a glycosylation-like mechanism that involves formation of an unusual glutamyl-ester between the E10 residue of the HCF-1_PRO_ repeat and the sugar molecule of UDP-GlcNAc (Lazarus et al. 2013). Indeed, using a model enzyme system unrelated to OGT:GlcNAc glycosylation, Kötzler and Withers (2016) have recently shown that glutamate glycosylation is sufficient to induce an autolysis of the peptide bond between the glutamic acid and the preceding residue, generating an N-terminal pyroglutamate on the C-terminal cleavage product (Kötzler and Withers 2016). If such a mechanism is also true for HCF-1_PRO_ repeat cleavage, our results here—in which even the Ser/Thr glycosylation-incompetent OGT:UDP-GlcNAc complexes are active for HCF-1_PRO_ repeat cleavage—suggest that such glutamate glycosylation is mechanistically distinct from Ser/Thr glycosylation. We hypothesize that this distinction lies in the nature of the critical acidic side chain of the E10 residue by which the carboxylate side chain group is positioned close to the sugar component of UDP-GlcNAc in the OGT Cat domain (Lazarus et al. 2013). Unlike for Ser/Thr glycosylation, this E10 residue probably does not require assisted deprotonation (for example, triggered by the nonbonded R_p_ oxygen of the UDP-GlcNAc α-phosphate) to activate the attack on the anomeric carbon of the UDP-GlcNAc sugar. The lack of such a preactivation step would explain why an OGT:UDP-GlcNAc complex incompetent for Ser/Thr glycosylation can still perform HCF-1_PRO_ repeat cleavage.

### How did OGT cleavage of HCF-1 arise?

HCF-1 proteolytic processing by OGT is unusual in the nature of both the cleavage signal, a large highly conserved HCF-1_PRO_ repeat sequence, and the enzyme responsible for cleavage (an enzyme otherwise only known to be involved in Ser/Thr glycosylation of intracellular proteins). Without knowledge of the enzyme responsible for HCF-1_PRO_ repeat cleavage, it was suggested that the HCF-1_PRO_ repeats appeared in HCF-1 proteins through a genetic recombination event, perhaps transposition, in a vertebrate progenitor (Capotosti et al. 2007). The suggestion was based on three observations: (1) As mentioned previously, only vertebrate HCF-1 proteins possess HCF-1_PRO_ repeats. (2) The HCF-1_PRO_ repeat sequence is highly conserved among and within vertebrate HCF-1 proteins but cannot be found even in degenerate forms elsewhere in vertebrate or nonvertebrate genomes. (3) As shown in Figure 7A, the multiple HCF-1_PRO_ repeat cleavage sites within individual HCF-1 proteins are generally encoded by one large exon as if they all appeared suddenly in an ancestral prevertebrate genome. We hypothesize here that, prior to its acquisition in an ancestral prevertebrate HCF-1-encoding gene by recombination, the HCF-1_PRO_ repeat sequence evolved in an ancient virus to serve as proteolytic processing sites for viral maturation.

We base this hypothesis on two observations. First, the sudden and highly conserved appearance of the HCF-1_PRO_ repeat sequence without any other related cellular sequence argues for an independent noncellular evolution of the HCF-1_PRO_ repeat sequence. Second, viruses often manipulate host cell machineries to their own end. With the knowledge that proteolytic maturation is a common feature of virus infectious cycles, we suggest here that an ancestral virus engineered a protein sequence (the HCF-1_PRO_ repeat) to manipulate the OGT:UDP-GlcNAc complex into promoting its own proteolytic maturation.

In principle, given the ability of a glycosylated glutamate residue to induce proteolysis (Kötzler and Withers 2016), a virus could have evolved the ability to be cleaved by OGT if it could create an appropriate OGT-binding site that positioned the key E10 glutamate to form a glycosyl-ester due to glycosylation. The HCF-1_PRO_ repeat exhibits both of these properties: First, it is tightly bound by OGT through the TPR domain, which, as shown here, is specifically an important feature of HCF-1_PRO_ repeat cleavage. In contrast, the HCF-1_PRO_ repeat cleavage region is not particularly favorable for HCF-1_PRO_ repeat binding to OGT, as the E10 residue hinders OGT binding, a strain that has been suggested to promote proteolysis (Bhuiyan et al. 2015). Second, as shown by an MD simulation (Supplemental Movie 1), the E10 residue is favorably positioned to induce an attack on the anomeric carbon of the UDP-GlcNAc sugar. However a virus may have evolved to be proteolytically cleaved by OGT, it would have targeted aspects of OGT function that are conserved even in organisms in which HCF-1_PRO_ repeat cleavage does not occur, as both *Trichoplax* and *Drosophila* OGTs can cleave the HCF-1_PRO_ repeat.

Development of a mechanism for proteolytic maturation via a glycosyltransferase whose activity is linked to the metabolic status of the cell could have been favorable for a virus, as it could permit the virus to coordinate its infectious cycle with cell metabolic status. We note that, before OGT cleavage of HCF-1 in vertebrate species evolved, HCF-1 cleavage was probably assured by Taspase 1 because vertebrate HCF-1 proteins retain vestigial nonfunctional Taspase 1 cleavage sites, as shown in Figure 7; a sudden acquisition of HCF-1_PRO_ repeats in the HCF-1 protein in vertebrates could have permitted an improved coordination of cell proliferation regulation with cell metabolism.

## Materials and methods

### Antibodies

Antibodies were purchased as follows: anti-GST 1-109 (sc-33613), anti-OGT (sc-32921), anti-CK2α (sc-12738), and anti-Nup62 (sc-48373) from Santa Cruz Biotechnology; anti*-O-*GlcNAc RL2 from Abcam; anti-T7 and anti-S tags from Novagen.; anti-Flag (F1804), anti-Actin, anti-*O-*GlcNAc (CTD110.6), and anti-His (H1029) from Sigma-Aldrich; and anti-HA (3F10) from Roche Applied Science.

### Bacterial and mammalian expression plasmids and recombinant protein purification

Creation of plasmids and DNA templates used in this study are described in the Supplemental Material along with the procedure for synthesis and purification of recombinant proteins from bacterial cells.

### In vitro HCF-1 cleavage and glycosylation assay

#### HCF-1rep1 cleavage and glycosylation

Purified HCF-1rep1 (2.5 μM) was incubated in the presence of 500–1000 nM OGT (unless otherwise noted) in 15 μL of TBS buffer (50 mM Tris at pH 7.4, 150 mM NaCl) supplemented with 1 mM UDP-GlcNAc (Sigma-Aldrich, U4375) and 1 mM DTT. The in vitro cleavage assay was incubated for 8 h at 37°C, after which the reaction was ended by the addition of 2× Laemmli buffer and sample boiling. Anti-GST and anti*-O-*GlcNAc RL2 antibodies were used to detect cleavage and glycosylation of HCF-1rep1, respectively. Anti-T7 and anti-OGT antibodies were used to determine levels of OGT.

#### HCF3R-SEE cleavage and glycosylation

HCF3R-SEE cleavage and glycosylation assay was performed by mixing 30 μM HCF3R-SEE and 2.0 μM OGT (wild type or mutant) in 30 μL of TBS buffer supplemented with 0.1 U/μL calf alkaline phosphatase, 1 mM UPD-GlcNAc, and 1 mM DTT for 4 h at 37°C. The reaction was ended by the addition of 2× Laemmli buffer and sample boiling. Anti-His and anti*-O-*GlcNAc RL2 antibodies were used to detect cleavage and glycosylation of HCF3R-SEE, respectively. Anti-OGT antibodies were used to determine levels of OGT.

### OGT assay

#### Nup62 glycosylation

Purified Nup62 (500 nM) was incubated in the presence of 500 nM OGT (wild type or mutant) in a reaction mix supplemented with 1 mM UPD-GlcNAc and 1 mM DTT for 90 min at 37°C. The reaction was terminated by the addition of 2× Laemmli buffer and sample boiling. Anti*-O-*GlcNAc RL2 was used to assess glycosylation of Nup62, while anti-S-tag and anti-T7 tags were used to detect total Nup62 and OGT protein levels, respectively.

#### CK2 glycosylation

CK2 (2500 U) purchased from New England Biolabs was incubated with 500 nM OGT (wild type or mutant) in 15 μL of TBS buffer supplemented with 1 mM UPD-GlcNAc and 1 mM DTT for 90 min at 37°C. The reaction was ended by the addition of 2× Laemmli buffer and sample boiling. Anti*-O-*GlcNAc RL2 was used to assess CK2 glycosylation, while anti-CK2 and anti-T7 tags were used to determine total CK2 and OGT protein levels, respectively.

### S_p_/R_p_-αS-UDP-GlcNAc cleavage and glycosylation assays

HCF-1rep1 cleavage and glycosylation and Nup62 glycosylation assays were performed as described above but with only 0.1 mM UDP-GlcNAc or S_p_/R_p_-αS-UDP-GlcNAc. Synthesis of S_p_/R_p_-αS-UDP-GlcNAc has been described previously (Schimpl et al. 2012).

### In vitro HCF-1–OGT-binding assay

GST-HCF-1rep1–OGT-binding assays were performed as described previously (Bhuiyan et al. 2015). Additional details are in the Supplemental Material.

### Immunoblotting

Proteins were resolved using SDS-PAGE gels and blotted onto nitrocellulose membranes. The following primary antibodies were used at 1:1000 dilution: anti-CK2α, anti-Nup62, anti-HA, and anti-His. The following primary antibodies were used at 1:2000 dilution: anti*-O-*GlcNAc RL2, anti*-O-*GlcNAc CTD110.6, and anti-OGT. The following primary antibodies were used at 1:5000 dilution: anti-Flag, anti-GST, and anti-Actin. The following primary antibodies were used at 1:10,000 dilution: anti-T7 and anti-S tag. IRDye 680 donkey anti-rabbit and IRDye 800 donkey anti-mouse antibodies were used at dilutions of 1:10,000. Blots were imaged using the Li-Cor Odyssey infrared imaging system (Li-Cor).

Uncleaved and cleaved HCF-1rep1 products were quantified using ImageJ software, and cleavage efficiencies were determined as the ratio of cleaved product band intensity over the precursor plus cleaved product band intensity. To quantify Nup62 *O-*GlcNAcylation in Figure 3C, relative band intensities were calculated by dividing the intensity of glycosylated Nup62 bands at the indicated time intervals by the intensity of glycosylated Nup62 after 60 min of assay.

### Molecular dynamics simulations and molecular modeling

A detailed description of molecular dynamic simulations and modeling performed in this study is in the Supplemental Material. Molecular graphics were generated using University of California at San Francisco Chimera (Pettersen et al. 2004).

### Sequence alignment

Sequences of OGT homologs from *Homo sapiens*, *Mus musculus*, *Gallus gallus, Anolis carolinensis*, *Danio rerio*, *D. melanogaster*, *Caenorhabditis elegans*, and *T. adhaerens* were aligned using ClustalW and edited and annotated with ALINE.

Detailed Materials and Methods with comprehensive details of UDP release measurements, bacterial protein purification, cell culture and transfections, in vitro binding assay, and molecular modeling and simulations are in the Supplemental Material.

## Supplementary Material

Supplemental Material
