# Supplementary material for: Proteolysis of HCF-1 by Ser/Thr glycosylation-incompetent O-GlcNAc transferase:UDP-GlcNAc complexes
Source: Genes Dev. 2016 Apr 15;30(8):960–72. doi: 10.1101/gad.275925.115 (PMC4840301; doi:10.1101/gad.275925.115)
Supplement: Supplemental Material [file supp_30_8_960__index.html]

Proteolysis of HCF-1 by Ser/Thr glycosylation-incompetent O-GlcNAc transferase:UDP-GlcNAc complexes — Proteolysis of HCF-1 by Ser/Thr glycosylation-incompetent O-GlcNAc transferase:UDP-GlcNAc complexes — Supplemental Material 

# Proteolysis of HCF-1 by Ser/Thr glycosylation-incompetent *O*-GlcNAc transferase:UDP-GlcNAc complexes

## Supplemental Material

- Supp\_Material.pdf - pdf file
- Supp\_Movie1.mov - mov file
