# Supplementary material for: Proteolysis of HCF-1 by Ser/Thr glycosylation-incompetent O-GlcNAc transferase:UDP-GlcNAc complexes
Source: Genes Dev. 2016 Apr 15;30(8):960–72. doi: 10.1101/gad.275925.115 (PMC4840301; doi:10.1101/gad.275925.115)
Supplement: Supplemental Material [file supp_gad.275925.115_Supp_Material.pdf]

Supplemental Materials and Methods

Bacterial expression plasmids

GST-HCF-1rep1 Vector: The generation of the GST–HCF-1rep1 construct (WT and E10A mutant) encoding HCF-1 amino acids 867-1071 has been previously described (Capotosti et al. 2011; Lazarus et al. 2013). Site-directed mutagenesis was used to generate the following GST–HCF-1rep1 mutants: H12A, H12P, E13A, E13P, H12P/E13P and T14A. The fusion protein containing N-Terminal GST-tag and C-terminal 6xHis-tag was purified from E. coli BL21 (DE3) as described previously (Lazarus et al. 2013; Bhuiyan et al. 2015).

Nus-S-His-Nup62 Vector: The human Nup62 cDNA Clone NM_012346 was purchased from Origene (SC321873) and used to amplify and clone Nup62 into the pET43.1 Ek/LIC vector according to the manufacturer's guidelines (Novagen) using the LIC-primers listed in Supplemental Table 1. The Nup62 fusion protein (~130 kDa) contained an N-terminal Nus-His-S tag and was purified as described below using Ni-Nta purification.

OGT Vectors: Mutants containing progressive OGT TPR deletions were expressed in the pET28-LIC vector. The pET28-LIC vector was created by inserting a previously described Pml1 restriction site containing the LIC-linker (Patel et al. 2012) into a pET28a vector linearized with BamH1 restriction enzyme. The resulting pET28-LIC vector was then digested with Pml1 to linearize the plasmid, followed by 3'-5' exonuclease treatment with T4 DNA Polymerase and dGTP to create 18-15 nucleotide overhangs as

previously described (Patel et al. 2012). To create progressive OGT TPR-deletion mutants, primers listed in Supplemental Table 1 were used to amplify N-terminal TPR deletion mutants of OGT. These PCR products were also treated with T4 DNA Polymerase and dCTP to generate pET28-LIC compatible, complementary overhangs. The LIC-treated vector and PCR products were then annealed and transformed into DH5α competent bacteria as previously described (Patel et al. 2012). The resulting OGT fusion proteins contain an N-terminal 6xHis and T7 tag.

The WT OGT expression vector pET24-NcOGT [gift from S. Walker, Harvard Medical School] was used as template to create internal OGT TPR-deletion mutants (Δ5–6, Δ7–8 and Δ9–10) using primers listed in Supplemental Table 1 and a site directed mutagenesis kit (Agilent). Single-point mutations of OGT were also made in the same construct using the primers listed in Supplemental Table 1.

The *D. melanogaster* OGT pET43-dOGT expression vector was a gift from J. Hanover, NIH. The human OGT pET43-hOGT expression vector was prepared by sub-cloning human OGT from pET24-NcOGT into a pET43.1 Ek/LIC vector according to the manufacturer's guidelines (Novagen) using the LIC-primers listed in Supplemental Table 1**.** pET43-dOGT and pET43-hOGT encoded proteins were synthesized as Nus-His-S tag fusion proteins and purified from BL21 bacteria as described below using Ni-Nta superflow resin (Qiagen). The WT and K815M Trichoplax OGTs purified as described previously(Selvan et al. 2015) were a kind gift of N. Selvan (University of Dundee).

HCF3R-SEE Vector: cDNA sequences coding for HCF3R-SEE were synthesized by Eurofins MWG Operon (Ebersberg, Germany) and cloned into pET47b vector digested

with BamH1 and Not1 using the primers listed in Supplemental Table 1. The fusion protein contained 8x-His tag at its N-terminus.

Mammalian expression plasmids

The plasmid construct encoding the N-terminal HA-tagged GST–HCF-1rep1 fusion protein (pCGN GST–HCF1rep1) has been previously described (Bhuiyan et al. 2015). DNA fragments encoding full-length OGT were PCR-amplified from their respective pET24–OGT vectors (WT/K396A/Swap/K842M) using the primers listed in Supplemental Table 1 and inserted into a Flag-tag pCGN derivative pCGF vector, using BamH1 restriction sites to create N-terminal Flag-tagged OGT expression plasmids (pCGF–OGT). All constructs were confirmed by sequencing.

Cell culture and plasmid transfections

HEK 293 cells were grown on 6-well plates at 37°C in DMEM with 10% FBS. For single plasmid transfection, cells were transfected with 1.0 µg of pCGF-OGT plasmid and 10 µl of Lipofectamine in an Opti-MEM medium according to the manufacturer's protocol (Invitrogen). For plasmid co-transfections, 1.0 µg of pCGN GST–HCF1rep1 plasmid was mixed with 0.5 µg of pCGF-OGT plasmid and transfected using 10 µl of Lipofectamine in Opti-MEM medium. 48 hours post-transfection, all cells were lysed in NP-40 lysis buffer (1% NP-40, 150 mM NaCl, 20 mM Tris pH 7.5) supplemented with Roche Complete protease inhibitors by incubation on ice for 15 min. The supernatant was clarified by spinning at 15,000 rcf for 15 min at 4°C. 20 µg of protein lysate was loaded from each sample and resolved using SDS-PAGE electrophoresis. Anti-HA antibody was used to

detect the cleavage of HA-HCF-1rep1 whereas anti-Flag and anti-OGT antibodies were used to detect the levels of OGT. Changes in overall protein *O*-GlcNAcylation were detected by immunoblotting with anti-*O*-GlcNAc antibodies (RL2 and CTD110.6).

Protein Purification

Transformed BL21 (DE3) bacteria were grown in typically 400 ml LB media containing appropriate antibiotics (Kanamycin for pET28-LIC, pET24, pET47b; Ampicillin for pET43) to an A600 optical density of 0.6, after which they were transferred to 16°C, induced with 0.2 mM IPTG and grown overnight at 16°C for 16 h. The bacteria were pelleted and re-suspended in 1:20 volume lysis buffer (TBS - 20 mM Tris [pH 7.4], 250 mM NaCl; supplemented with 1 mM PMSF and 0.1 mg/ml lysozyme (Roche Applied Science)) for 30 min on ice. The lysate was then sonicated and centrifuged at 60,000 RCF in a Beckman SS-34 rotor for 1 h at 4°C. Imidazole was added to the clarified supernatant to a final concentration of 25 mM before the lysate was incubated with Ni-NTA agarose superflow resin for 1 hr at 4°C, which was prewashed with TBS + 40 mM imidazole for nickel-affinity purification. After incubation, the flow through was removed and the resin washed with 3 column volumes of lysis buffer + 40 mM imidazole. The fusion protein was then eluted in TBS + 250 mM imidazole. To concentrate and desalt the eluate, Amicon concentration tubes (Millipore) were used as per the manufacturer's instructions. The concentrated protein was supplemented with 1 mM DTT and stored at -80°C.

Note that the GST–HCF-1rep1 fusion protein co-purifies with a contaminating protein that contains a 93-amino acid N-terminal GST-truncation, owing to an internal

translation initiation at GST M94. This uncleaved truncated GST–HCF-1rep1 protein nearly co-migrates with the OGT-mediated cleavage product of HCF-1rep1; the cleaved truncated GST-HCF-1rep1 protein migrates faster and is generally not observed under our PAGE conditions. To overcome this contaminant, we take advantage of an anti-GST (1-109) antibody, which allows us to analyze HCF-1rep1 cleavage assays with cleaner backgrounds. Nevertheless, the glycosylated product of this N-truncated GST–HCF-1rep1 protein is still detectable in blotting with anti-*O*-GlcNAc antibodies particularly if the OGT used in assay is defective for proteolysis but not glycosylation, as seen in Figure 2B lane 4 (marked with asterisk) and in Figure 3B lane 3 co-migrating with the highly glycosylated cleaved HCF-1rep1 molecules.

In vitro HCF-1–OGT binding assay

GST–HCF-1rep1–OGT pull-down assays were performed as described previously (Bhuiyan et al. 2015). Prior to the OGT pull-down, 20 µl of a slurry of α-T7 antibody-conjugated beads (goat polyclonal, Abcam) was incubated with PBS containing 5% (w/v) bovine serum albumin (BSA) for 1 h at 4°C to decrease non-specific binding of GST–HCF-1rep1 to the agarose beads. Subsequently, the beads were washed extensively in PBS. For the OGT pull-down, 2.5 µg GST–HCF-1rep1 and 5 µg of OGT were pre-incubated in 0.5% NP40 buffer (0.5% NP40, 10 mM Tris-Cl pH 8.0, 150 mM NaCl) supplemented with 5 mM DTT, in a rotating incubator for 1 h at 20°C. After incubation, 10% of the reaction was removed as an input control for the OGT pull-down. The washed α-T7 agarose beads were added to the reaction, the volume increased to 500 µl using NP40 buffer and the suspension incubated for 1 h at 20°C. The beads were

centrifuged and subsequently washed in NP40 buffer at least three times for 5 min each time at room temperature and the GST–HCF-1rep1–OGT complexes eluted by boiling for 5 min in 20 µl of 2X Laemmli buffer. HCF-1rep1–OGT binding was subsequently analyzed by SDS-PAGE followed by immunoblot.

UDP-release measurement

UDP generation was determined in reactions containing 100 nM WT OGT in 50 mM Tris-HCl, pH 7.5, 10 µM sodium dithionate and 15 uM of substrate protein (HCF-1rep1, HCF-1rep1E10Q) in a total volume of 100 µl. Reaction mixtures were pre-incubated for 15 min and initiated by addition of R_p-αS-UDP-GlcNAc to a final concentration of 25 µM. The reaction was stopped after 6 hr by addition of 200 µl stop buffer (25 mM HEPES, pH 7.4, 10 mM NaCl, 50% (v/v) MeOH, 25 µM fluorophore — a UDP-sensitive xanthene-based Zn(II) complex described previously (Borodkin et al. 2014) — and 40 µM pyrocatechol violet). UDP release was detected fluorimetrically on a fluorescence Microplate reader at excitation and emission wavelengths of 485 nm and 530 nm, respectively. Background fluorescence of protein samples was substracted from the UDP consumption measurements. No OGT-only mediated UDP-GlcNAc hydrolysis could be observed during the course of the assay.

Molecular Modeling of Full-Length OGT Structure and Molecular Graphics

The 3D structure of the full-length human OGT was reconstructed from the experimental structures of the superhelical TPR domain (residues 13-400, PDB ID 1W3B (Jinek et al. 2004)) and of the Cat domain (residues 313-1028; PDB ID 4N3B (Lazarus et al. 2011)).

The reconstruction was performed using the Modeller Program (Sali and Blundell 1993; Eswar et al. 2007). 500 models were generated by satisfaction of spatial restraints through minimization and simulated annealing, and the model with the best Modeller objective function was retained. Molecular graphics were generated using UCSF Chimera (Pettersen et al. 2004).

Molecular Dynamics Simulations

Periodic-boundary MD simulations were carried out with GROMACS Version 4.6.5 (Hess et al. 2008; Bjelkmar et al. 2010) using the all-atom CHARMM27 force field (MacKerell et al. 1998; Mackerell et al. 2004) and the TIP3P water model (Jorgensen et al. 1983). Electrostatic interactions were calculated with the Ewald particle-mesh method (Essmann et al. 1995) with a grid spacing of 1.2 Å and a spline interpolation of order 4. A cutoff of 10 Å was applied for the real-space direct sum part of the Ewald sum, and a cutoff of 14 Å for the van der Waals interactions. Dispersion corrections were applied to the energy. Bonds involving hydrogen atoms were constrained using the P-LINCS algorithm (Hess et al. 2008). The system was coupled to a barostat (Berendsen et al. 1984) with a relaxation time of 1 ps. The solute and the solvent were separately coupled to two thermostats (Bussi et al. 2007), each with a relaxation time of 0.2 ps. The time integration step was set to 2 fs, the temperature to 300 K, and the pressure to 1 bar. A cubic simulation cell with an edge length of 120 Å was used to prevent direct interactions between periodic images, resulting in about 170,000 atoms per system. Sodium atoms were added to neutralize each system. Initial structures were optimized, heated from 0 to 300 K over a period of 0.1 ns, equilibrated for a further 0.9 ns

restraining each solute non-hydrogen atom to its original position, and finally equilibrated for 0.5 ns without restraints before data collection. For each system, 8 simulations were carried out for a total of 40 ns of production time, saving coordinates every 0.05 ns.

OGT simulations in complex with HCF-1_{PRO}-repeat 2 (residues 5-24) were based on the X-ray structure 4N3B (Lazarus et al. 2013), replacing UDP-5S-GlcNAc with UDP-GlcNAc and Q10 with E10. When replacing Q10 by E10, we observed that neutralizing its side chain carboxylate group was necessary to obtain a stable OGT-HCF-1 complex. The added proton formed a stable hydrogen bond with the backbone oxygen of G654.

Two different systems were simulated: HCF-1_{PRO}-repeat 2 with UDP-GlcNAc and wild-type OGT or OGT D554H_H558D Swap. For analysis, structural snapshots were superimposed either based on the backbone of the OGT Cat domain (residues 467-1028) or TPR domain (residues 312-466) before calculating backbone root mean square deviations (RMSD) from the respective X-ray structure and root mean square fluctuations (RMSF) around the average structure.

Supplemental references

Berendsen HJC, Postma JPM, van Gunsteren WF, DiNola A, Haak JR. 1984. Molecular dynamics with coupling to an external bath. *The Journal of Chemical Physics* 81: 3684-3690.

Bhuiyan T, Waridel P, Kapuria V, Zoete V, Herr W. 2015. Distinct OGT-Binding Sites Promote HCF-1 Cleavage. *PLoS One* 10: e0136636.

Bjelkmar P, Larsson P, Cuendet MA, Hess B, Lindahl E. 2010. Implementation of the CHARMM Force Field in GROMACS: Analysis of Protein Stability Effects from Correction Maps, Virtual Interaction Sites, and Water Models. *Journal of Chemical Theory and Computation* 6: 459-466.

Borodkin VS, Schimpl M, Gundogdu M, Rafie K, Dorfmueller HC, Robinson DA, van Aalten DM. 2014. Bisubstrate UDP-peptide conjugates as human O-GlcNAc transferase inhibitors. *Biochem J* 457: 497-502.

Bussi G, Donadio D, Parrinello M. 2007. Canonical sampling through velocity rescaling. *The Journal of Chemical Physics* 126: 014101.

Capotosti F, Guernier S, Lammers F, Waridel P, Cai Y, Jin J, Conaway JW, Conaway RC, Herr W. 2011. O-GlcNAc transferase catalyzes site-specific proteolysis of HCF-1. *Cell* 144: 376-388.

Essmann U, Perera L, Berkowitz ML, Darden T, Lee H, Pedersen LG. 1995. A smooth particle mesh Ewald method. *The Journal of Chemical Physics* 103: 8577-8593.

Eswar N, Webb B, Marti-Renom MA, Madhusudhan MS, Eramian D, Shen MY, Pieper U, Sali A. 2007. Comparative protein structure modeling using MODELLER. *Curr Protoc Protein Sci* Chapter 2: Unit 2 9.

Hess B, Kutzner C, van der Spoel D, Lindahl E. 2008. GROMACS 4: Algorithms for Highly Efficient, Load-Balanced, and Scalable Molecular Simulation. *Journal of Chemical Theory and Computation* 4: 435-447.

Jinek M, Rehwinkel J, Lazarus BD, Izaurralde E, Hanover JA, Conti E. 2004. The superhelical TPR-repeat domain of O-linked GlcNAc transferase exhibits structural similarities to importin alpha. *Nature structural & molecular biology* 11: 1001-1007.

Jorgensen WL, Chandrasekhar J, Madura JD, Impey RW, Klein ML. 1983. Comparison of simple potential functions for simulating liquid water. *The Journal of Chemical Physics* 79: 926-935.

Lazarus MB, Jiang J, Kapuria V, Bhuiyan T, Janetzko J, Zandberg WF, Vocadlo DJ, Herr W, Walker S. 2013. HCF-1 is cleaved in the active site of O-GlcNAc transferase. *Science* 342: 1235-1239.

Lazarus MB, Nam Y, Jiang J, Sliz P, Walker S. 2011. Structure of human O-GlcNAc transferase and its complex with a peptide substrate. *Nature* 469: 564-567.

MacKerell AD, Bashford D, Bellott M, Dunbrack RL, Evanseck JD, Field MJ, Fischer S, Gao J, Guo H, Ha S et al. 1998. All-Atom Empirical Potential for Molecular Modeling and Dynamics Studies of Proteins. *The Journal of Physical Chemistry B* 102: 3586-3616.

Mackerell AD, Feig M, Brooks CL. 2004. Extending the treatment of backbone energetics in protein force fields: Limitations of gas-phase quantum mechanics in reproducing

protein conformational distributions in molecular dynamics simulations. *Journal of computational chemistry* 25: 1400-1415.

Patel A, Muñoz A, Halvorsen K, Rai P. 2012. Creation and validation of a ligation-independent cloning (LIC) retroviral vector for stable gene transduction in mammalian cells. *BMC biotechnology* 12: 3.

Pettersen EF, Goddard TD, Huang CC, Couch GS, Greenblatt DM, Meng EC, Ferrin TE. 2004. UCSF Chimera--a visualization system for exploratory research and analysis. *Journal of computational chemistry* 25: 1605-1612.

Sali A, Blundell TL. 1993. Comparative protein modelling by satisfaction of spatial restraints. *J Mol Biol* 234: 779-815.

Selvan N, Mariappa D, van den Toorn HW, Heck AJ, Ferenbach AT, van Aalten DM. 2015. The Early Metazoan Trichoplax adhaerens Possesses a Functional O-GlcNAc System. *The Journal of biological chemistry* 290: 11969-11982.

Supplemental Figure Legends

Supplemental Figure 1. 'THE' linker region.

(A.) Structural representation of thermal fluctuations (small and blue=rigid; large and red=flexible) in the OGT:UDP-GlcNAc complex-bound HCF-1_{PRO}-repeat 2 shows that the flexible 'THE' linker region (arrow) is situated at the interface of the OGT Cat and TPR domains.

(B.) 'THE' linker-region mutations do not impact the OGT mediated *O*-GlcNAcylation of HCF-1rep1 (WT or mutant). Levels of *O*-GlcNAcylated HCF-1rep1 proteins were detected using the anti-*O*-GlcNAc RL2 antibody.

(C.) The mutations within the HCF-1_{PRO}-repeat 'THE' linker region do not inhibit the affinity of HCF-1rep1 protein toward OGT. The binding assay was performed as described in Materials and Methods. The previously described OGT affinity impaired HCF-1rep1 T14A mutant (Bhuiyan et al. 2015) was used as a positive control for detection of debilitated OGT binding.

(▭, glycosylated un-cleaved HCF-1rep1 substrate, ○, glycosylated cleaved product.)

Supplemental Figure 2. Nup62 glycosylation and OGT autoglycosylation assays performed using OGT TPR-deletion mutants.

(A.) Mutant OGT's containing progressive N-terminal TPR deletions were incubated with Nup62 and glycosylation assayed as described in Materials and Methods. Nup62 glycosylation and OGT autoglycosylation were detected using the anti-*O*-GlcNAc RL2

antibody (top panel), whereas anti-Nup62 and anti-T7 antibodies were used to detect the NUS-S-His-Nup62 fusion and OGT proteins (lower panels), respectively. Right, schematic illustration of progressive deletions.

(B.) Mutant OGT's containing internal deletions of two TPR motifs at a time were incubated with Nup62 and assayed for substrate glycosylation as described in (A.). Right, Schematic illustration of internal deletions with deletion start- and end-points indicated. Note that the upper portions of the top panels in A and B showing Nup62 glycosylation are identical to those shown in Figure 2A and B, respectively.

(C.) A sequence alignment of paired OGT TPRs 1-12 adapted from Jinek et. al (2004) was prepared using a web-based Multi-sequence align tool (http://www.bioinformatics.org/SMS/multi_align.html). Identical amino-acids present in 50% of the aligned sequences are shaded in blue and similar amino-acids are like-wise shaded yellow. Solvent exposed residues in each TPR are marked with asterisks.

Supplemental Figure 3. K396A OGT glycosyltransferase and proteolytic-activity.

(A.) Nup62 was incubated with WT or mutant OGT's to assay Nup62 glycosylation. Anti-*O*-GlcNAc RL2 antibody was used to detect Nup62 glycosylation, whereas anti-OGT and anti-Nup62 antibodies were used to detect OGT and Nup62 protein levels, respectively. Note that the top panel showing Nup62 glycosylation is identical to the one shown in Figure 3B.

(B.) In vitro HCF-1rep1, and WT and mutant OGT binding analysis was performed as described in Materials and Methods. Anti-GST and anti-OGT antibodies were used to detect HCF-1rep1 and OGT protein levels, respectively.

(C.) Nup62 was incubated with either WT or K396A OGT for indicated time periods and assayed for glycosylation as described in (A.).

(D.) HCF-1rep1 was incubated with either WT or K396A OGT for indicated time periods and assayed for HCF-1rep1 cleavage and glycosylation as described in Materials and Methods.

(▬, un-cleaved HCF-1rep1 substrate, ●, cleaved product.)

(▭, glycosylated un-cleaved HCF-1rep1 substrate, ○, glycosylated cleaved product.)

<u>**Supplemental Figure 4. Glycosylation and HCF-1 proteolytic activities of OGT mutants.**</u>

(A.) WT or mutant OGT's were incubated with CK2 substrate as described in Materials and Methods. CK2 Glycosylation was assessed with the anti-*O*-GlcNAc RL2 antibody, whereas anti-OGT and anti-CK2 were used to detect corresponding OGT and CK2 protein levels. A long exposure of the CK2 glycosylation assay is shown (High Intensity) to emphasize the lack of OGT D554H_H558A Swap mutant glycosylation activity in this assay.

(B.) MD simulations of WT OGT and the OGT D554H_H558D Swap mutant bound to HCF-1_{PRO}-repeat 2 show that the mutagenesis does not perturb the OGT structure, as demonstrated by comparing backbone RMSD values of the two simulations.

(C.) HCF-1rep1 cleavage and glycosylation in the presence of WT and OGT TPR- (lanes 3–5) and Cat- (lanes 6–8 and 11–16) domain mutants were assayed with anti-GST and anti-*O*-GlcNAc RL2 antibody, respectively.

(▬, un-cleaved HCF-1rep1 substrate, ●, cleaved product.)

(▭, glycosylated un-cleaved HCF-1rep1 substrate, ○, glycosylated cleaved product.)

Supplemental Figure 5. Swap OGT mutant fails to enhance endogenous protein *O*-GlcNAcylation.

HEK293 cells were left untransfected (–) or transfected with different Flag-OGT expression vectors as described in Materials and Methods. Protein *O*-GlcNAcylation in HEK293 cells was visualized by *O*-GlcNAc blot using either anti-*O*-GlcNAc RL2 or anti-*O*-GlcNAc CTD110.6 antibody. Levels of ectopic OGT were detected using anti-Flag antibody and both endogenous and ectopic OGT with OGT antibody. Anti-actin antibody was used to detect actin protein as loading control.

Supplemental Figure 6. Sequence conservation among OGT protein homologs found in different organisms.

Sequence conservation among OGT protein homologs found in different organisms (human, mouse, chicken, lizard, zebra-fish, worm, placozoa and fly) is shown. The blue bar represents the TPR domain whereas the tan bar represents the Cat domain of human OGT. TPR domain residues which make contacts with HCF-1_{PRO}-repeat 2 (backbone or side-chain) are marked with inverted yellow triangles. The TPR 12 residue

K396 is marked with a green box and the N-Cat domain D554 and H558 residues are marked with a red dot and boxed. Analyzed C-Cat domain residues, which surround the UDP-GlcNAc binding site, are marked with red stars (K842, F868, K898, H901, D925).

<u>Supplemental Movie 1</u>

Simulation of E10 residue of HCF-1$_{PRO}$-repeat attack on the UDP-GlcNAc sugar molecule.

The movie illustrates the S_N2-like attack of the glutamate's carboxylate on the anomeric carbon of the UDP-GlcNAc sugar. It was obtained by energy minimization in a QM/MM description. The quantum mechanics (QM) part contains residues C9 and E10 of the HCF-1$_{PRO}$ repeat, as well as the sugar of UDP-GlcNAc. During the minimization, we forced one oxygen atom of the glutamate closer to the anomeric carbon of the sugar. We observed a spontaneous separation of the phosphate from the anomeric carbon, while the latter was getting planar, in a S_N2-like mechanism. The minimization was performed with the CHARMM program (PMID: 19444816), version c36b1, using SCC-DFTB for the QM part and the CHARMM27 force field (PMID: 24889800, DOI: 10.1002/(SICI)1096-987X(20000130)21:2<86::AID-JCC2>3.0.CO;2-G) for the classical (MM) part.

SUPPLEMENTAL FIGURE 1

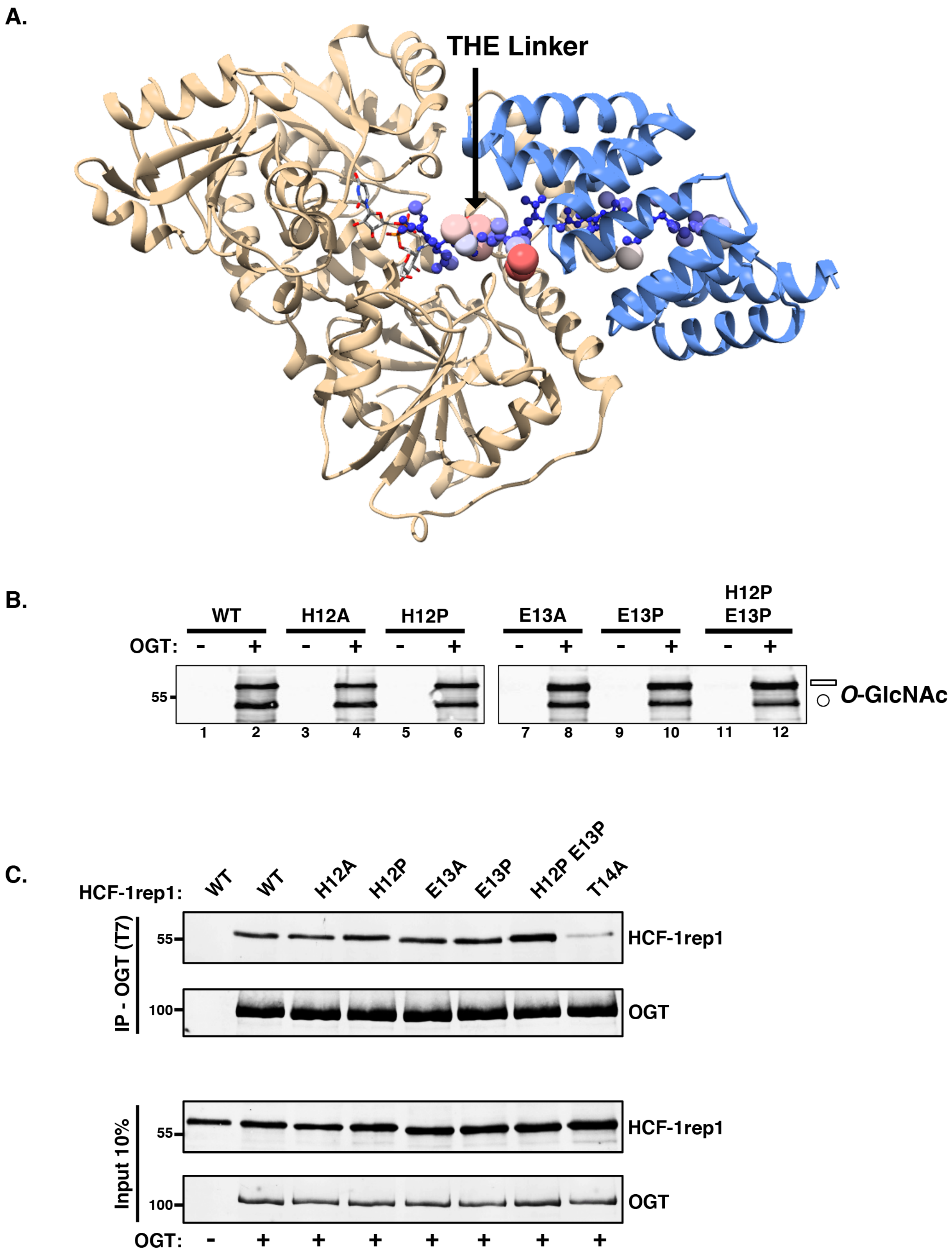

SUPPLEMENTAL FIGURE 2

A.

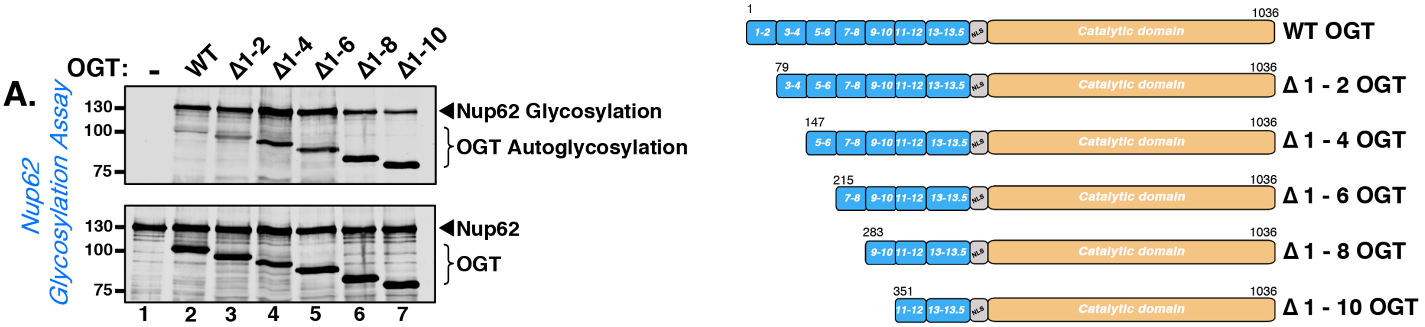

B.

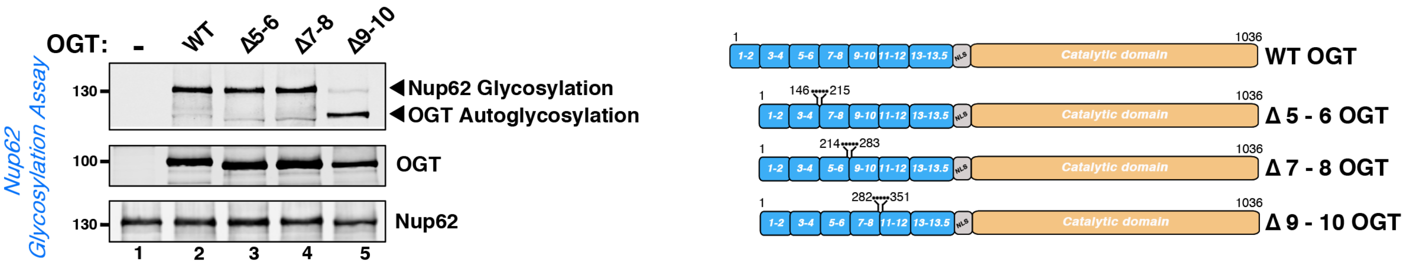

C.

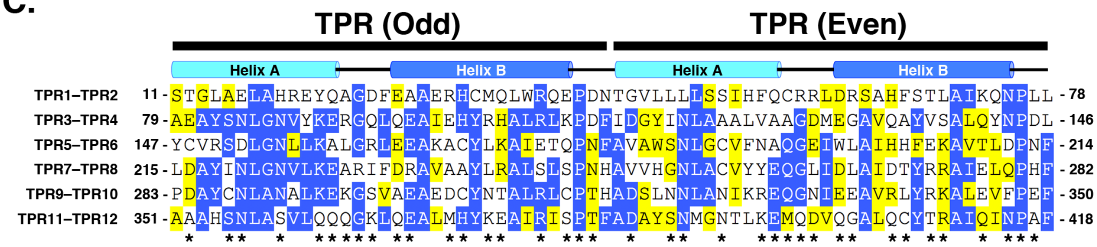

SUPPLEMENTAL FIGURE 3

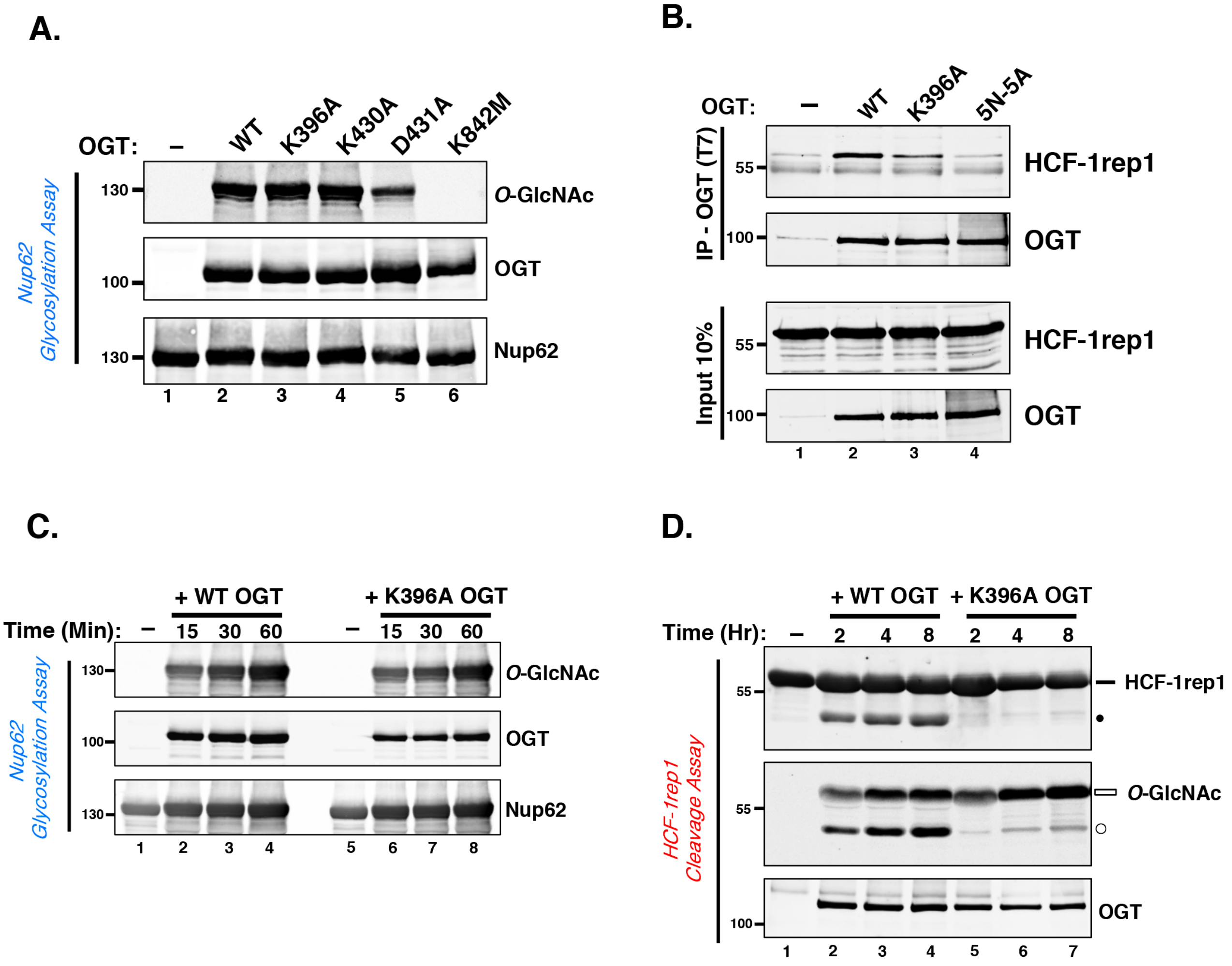

SUPPLEMENTAL FIGURE 4

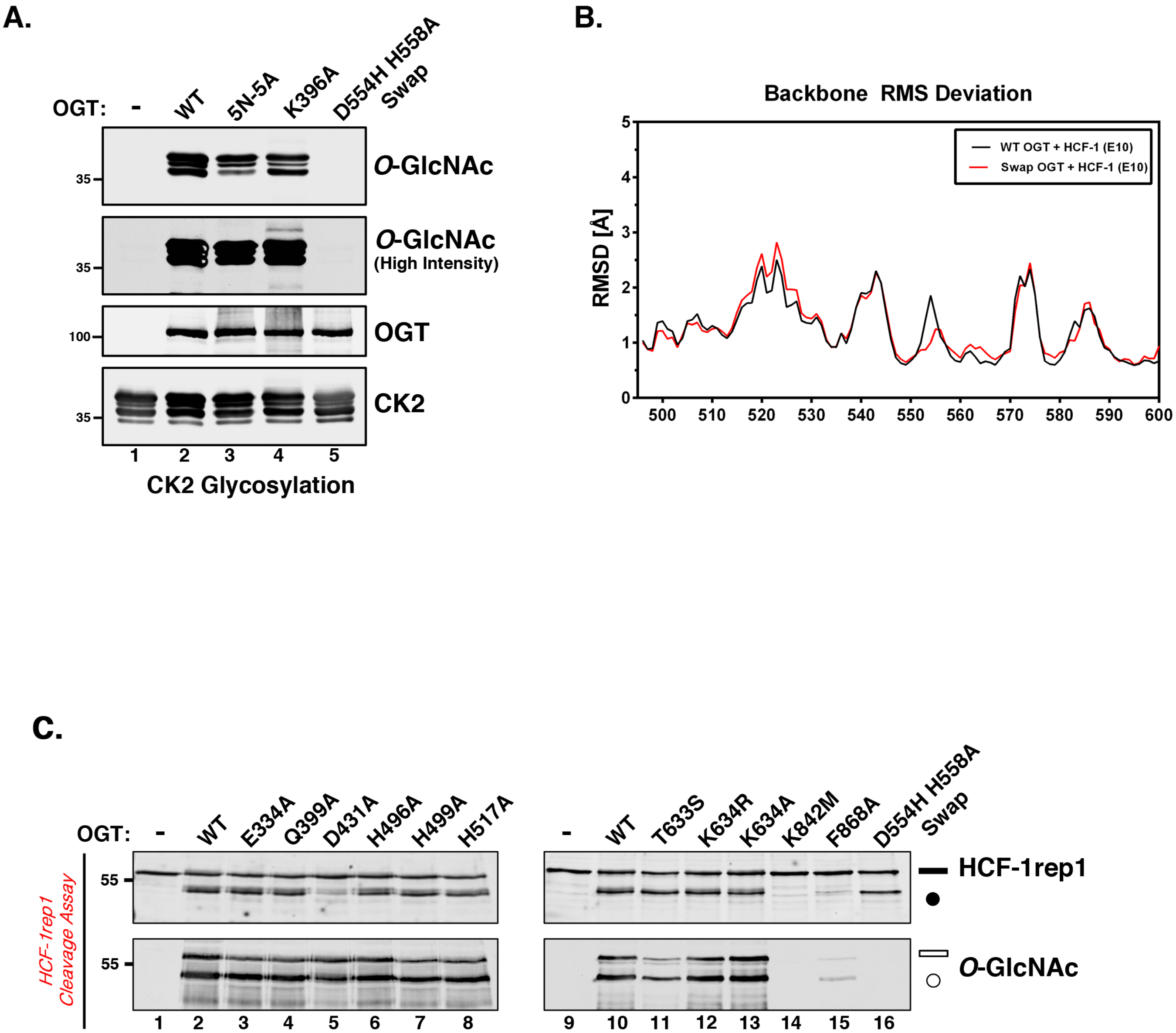

SUPPLEMENTAL FIGURE 5

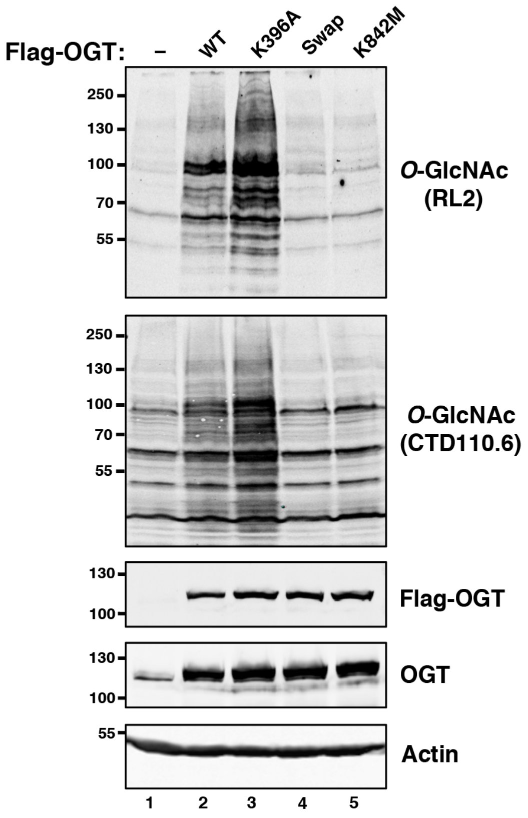

SUPPLEMENTAL FIGURE 6

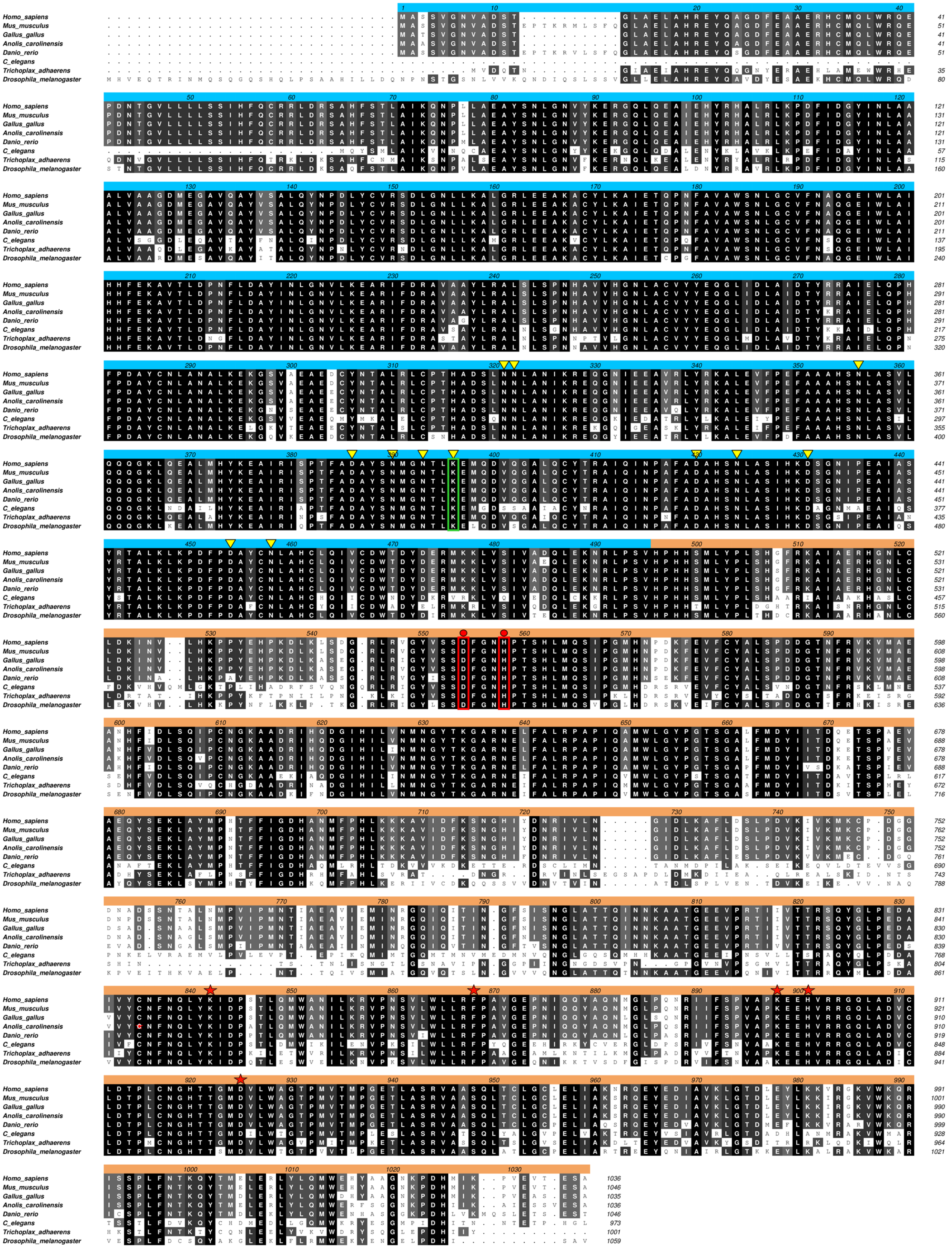

Supplemental Table 1

Mutant	Expression Vector	Forward Primer	Reverse Primer
E334A	pET24-Nc OGT	CGA GAA CAG GGA AAC ATT GCA GAG GCA GTT CGC TTG TAT	ATA CAA GCG AAC TGC CTC TGC AAT GTT TCC CTG TTC TCG
K396A	pET24-Nc OGT	GGA AAC ACT CTA GCT GAG ATG CAG GAT GTT CAG GGA GCC	CAT CCT GCA TCT CAG CTA GAG TGT TTC CCA TAT TAG AG
Q399A	pET24-Nc OGT	AAC ACT CTA AAG GAG ATG GCT GAT GTT CAG GGA GCC TTG	CAA GGC TCC CTG AAC ATC AGC CAT CTC CTT TAG AGT GTT
K430A	pET24-Nc OGT	AAT CTG GCT TCC ATT CAT GCG GAT TCA GGG AAT ATT CCA	TGG AAT ATT CCC TGA ATC CGC ATG AAT GGA AGC CAG ATT
D431A	pET24-Nc OGT	CTG GCT TCC ATT CAT AAG GCT TCA GGG AAT ATT CCA GAA	TTC TGG AAT ATT CCC TGA AGC CTT ATG AAT GGA AGC CAG
H496A	pET24-Nc OGT	AAT AGG TTG CCT TCT GTG GCT CCT CAT CAT AGT ATG CTA	TAG CAT ACT ATG ATG AGG AGC CAC AGA AGG CAA CCT ATT
H499A	pET24-Nc OGT	CCT TCT GTG CAT CCT CAT GCT AGT ATG CTA TAT CCT CTT	AAG AGG ATA TAG CAT ACT AGC ATG AGG ATG CAC AGA AGG
H517A	pET24-Nc OGT	AAG GCT ATT GCT GAG AGG GCC GGC AAC CTG TGC TTA GAT	ATC TAA GCA CAG GTT GCC GGC CCT CTC AGC AAT AGC CTT
S553A	pET24-Nc OGT	CGT GTA GGA TAT GTG AGT GCC GAC TTT GGG AAT CAT CCT	AGG ATG ATT CCC AAA GTC GGC ACT CAC ATA TCC TAC ACG
D554A	pET24-Nc OGT	GTA GGA TAT GTG AGT TCC GCC TTT GGG AAT CAT CCT ACT	AGT AGG ATG ATT CCC AAA GGC GGA ACT CAC ATA TCC TAC
H558A	pET24-Nc OGT	AGT TCC GAC TTT GGG AAT GCT CCT ACT TCT CAC CTT ATG	CAT AAG GTG AGA AGT AGG AGC ATT CCC AAA GTC GGA ACT
D554A H558A	pET24-Nc OGT	GTG AGT TCC GCC TTT GGG AAT GCT CCT ACT TCT CAC CTT ATG C	GAA GTA GGA GCA TTC CCA AAG GCG GAA CTC ACA TAT CCT AC
SWAP	pET24-Nc OGT	GTA GGA TAT GTG AGT TCC CAC TTT GGG AAT GAT CCT ACT TCT CAC	CAT AAG GTG AGA AGT AGG ATC ATT CCC AAA GTG GGA ACT CAC ATA TCC TAC
T633S	pET24-Nc OGT	CTA TAG TAA GGG CGC TCG AAA TGA GCT TTT TGC TCT C	CTT ACT ATA GCC ATT CAT ATT TAC AAG GAT ATG AAT TCC
K634R	pET24-Nc OGT	ACT AGG GGC GCT CGA AAT GAG CTT TTT GCT CTC AGG	GCC CCT AGT ATA GCC ATT CAT ATT TAC AAG GAT ATG
K634A	pET24-Nc OGT	ACT GCG GGC GCT CGA AAT GAG CTT TTT GCT CTC AGG	GCC CGC AGT ATA GCC ATT CAT ATT TAC AAG GAT ATG
K842M	pET24-Nc OGT	AAC TTT AAT CAG TTG TAT ATG ATT GAC CCT TCT ACT TTG	CAA AGT AGA AGG GTC AAT CAT ATA CAA CTG ATT AAA GTT
F868A	pET24-Nc OGT	GTA CTC TGG CTG TTG CGT GCT CCA GCA GTA GGA GAA CCT	AGG TTC TCC TAC TGC TGG AGC ACG CAA CAG CCA GAG TAC
K898A	pET24-Nc OGT	GTT GCT CCT GCA GAG GAA CAC GTC AGG AGA GGC	GTG TTC CTC TGC AGG AGC AAC AGG TGA AAA AAT GAT ACG
H901A	pET24-Nc OGT	AAA GAG GAA GCC GTC AGG AGA GGC CAG CTG GCT GAT G	CTC TCC TGA CGG CTT CCT CTT TAG GAG CAA CAG G
H OGT-pET28-LIC Primers	pET28-LIC	CAC ACC ATC ATC TCA CGA TGG CGT CTT CCG TGG GCA AC	CTC ACA TTA TTC CAC GTT ATG CTG ACT CAG TGA CTT CAA
Δ 1-2	pET28-LIC	CAC ACC ATC ATC TCA CGG CAG AAG CTT ATT CGA ATT TGG	H OGT-pET28-LIC Reverse Primer
Δ 1-4	pET28-LIC	CAC ACC ATC ATC TCA CGT ACT GTG TTC GCA GTG ACC TG	H OGT-pET28-LIC Reverse Primer
Δ 1-6	pET28-LIC	CAC ACC ATC ATC TCA CGC TGG ATG CTT ATA TCA ATT TAG	H OGT-pET28-LIC Reverse Primer
Δ 1-8	pET28-LIC	CAC ACC ATC ATC TCA CGC CTG ATG CTT ACT GCA ACC TAG	H OGT-pET28-LIC Reverse Primer
Δ 1-10	pET28-LIC	CAC ACC ATC ATC TCA CGG CTG CTG CCC ATT CAA ATT TAG	H OGT-pET28-LIC Reverse Primer
Δ 5-6	pET24-Nc OGT	CAG TAC AAT CCT GAT TTG CTG GAT GCT TAT ATC AAT	ATT GAT ATA AGC ATC CAG CAA ATC AGG ATT GTA CTG
Δ 7-8	pET24-Nc OGT	ACC CTT GAC CCA AAC TTT CCT GAT GCT TAC TGC AAC	GTT GCA GTA AGC ATC AGG AAA GTT TGG GTC AAG GGT

Δ 9-10	pET24-Nc OGT	GAA CTA CAA CCA CAT TTC GCT GCT GCC CAT TCA AAT	ATT TGA ATG GGC AGC AGC GAA ATG TGG TTG TAG TTC
H OGT-pET43-LIC Primers	pET43.1-Ek/LIC	GAC GAC GAC AAG ATC GCG TCT TCC GTG GGC	GAG GAG AAG CCC GGT TAT GCT GAC TCA GTG ACT TC
Nup62-pET43-LIC Primers	pET43.1-Ek/LIC	GAC GAC GAC AAG ATG AGC GGG TTT AAT TTT	GAG GAG AAG CCC GGT CAG TCA AAG GTG ATC CGG A
HCF3R Cloning Primers	pET47b	CCA GGA TCC GCC GGG TAC CGT TAC CCT GG	CTG CGG CCG CTT AAT TAG CTC CAA CAG AAG ACA TAG C
H OGT pCGF Primers	pCGF	CTA GGA TCC ATG GCG TCT TCC GTG GGC AAC	GAT GAT GGA TCC TTA TGC TGA CTC AGT GAC TTC AAC
